# Supplementary material for: Structural and biochemical insights into the mechanism of the Gabija bacterial immunity system
Source: Nat Commun. 2024 Jan 29;15:836. doi: 10.1038/s41467-024-45173-7 (PMC10822852; doi:10.1038/s41467-024-45173-7)
Supplement: Supplementary file 1 — Supplementary Information [file 41467_2024_45173_MOESM1_ESM.pdf]

Supplementary Information

Structural and biochemical insights into the mechanism of the Gabija bacterial immunity system

Yanwu Huo[1, *, #], Lingfei Kong[1, 2, #], Ye Zhang[1, 2, #], Min Xiao[1], Kang Du[1], Sunyuntao Xu[1], Xiaoxue Yan[1], Jun Ma[3, *] and Taotao Wei[1, 2, *]

[1] National Laboratory of Biomacromolecules, Institute of Biophysics, Chinese Academy of Sciences, 15 Datun Road, Chaoyang District, Beijing 100101, China

[2] School of Biological Sciences, University of Chinese Academy of Sciences, 19 Yuquan Road, Shijingshan District, Beijing 100049, China

[3] Institute of Infectious Diseases, Shenzhen Bay Laboratory, Gaoke Innovation Center, Guangqiao Road, Guangming District Shenzhen, Guangdong, 518132, China

[#] These authors contributed equally.

* Correspondence: huoyw@ibp.ac.cn (YH), majun@szbl.ac.cn (JM) and weitt@ibp.ac.cn (TW)

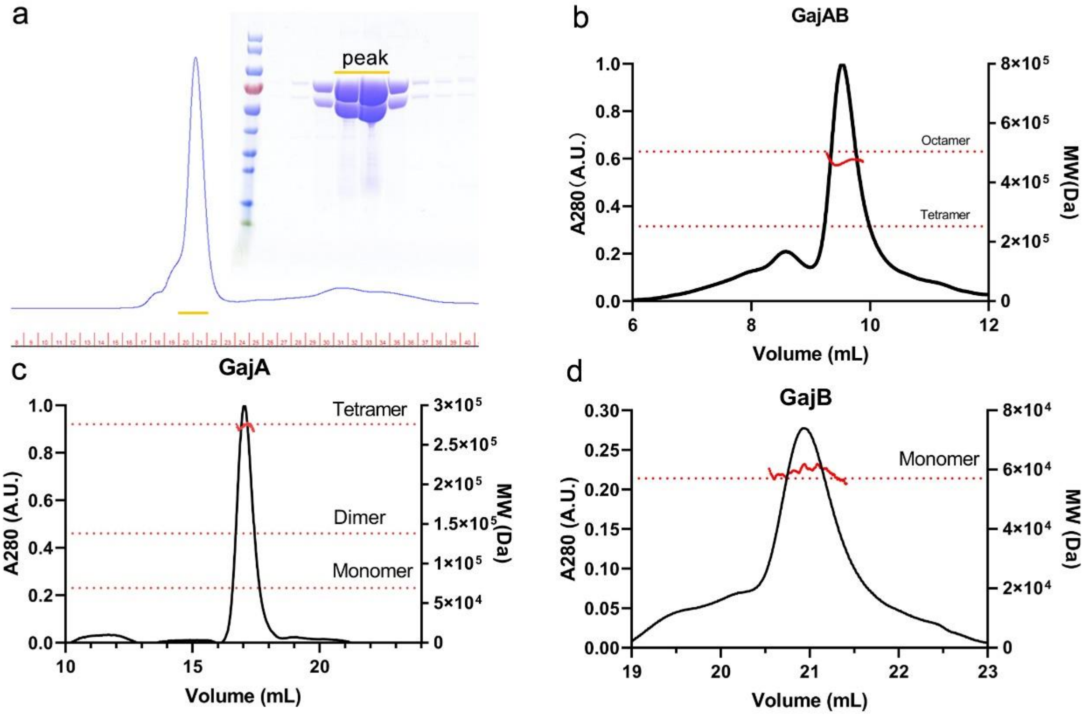

Supplementary Fig.1 Composition analysis of Gabija complex. a, Elution profile of the Gabija complex on an analytical Superdex 200 10/300 size exclusion column and analysis of the fractions using the Coomassie Blue-stained SDS-PAGE. Peak position is marked with yellow line both in the SDS-PAGE and Elution profile. (**b**, **c**, **d**), SEC-MALS analysis of Gabija complex , GajA and GajB respectively. UV trace (black) and calculated molecular weight based on light scattering are shown.

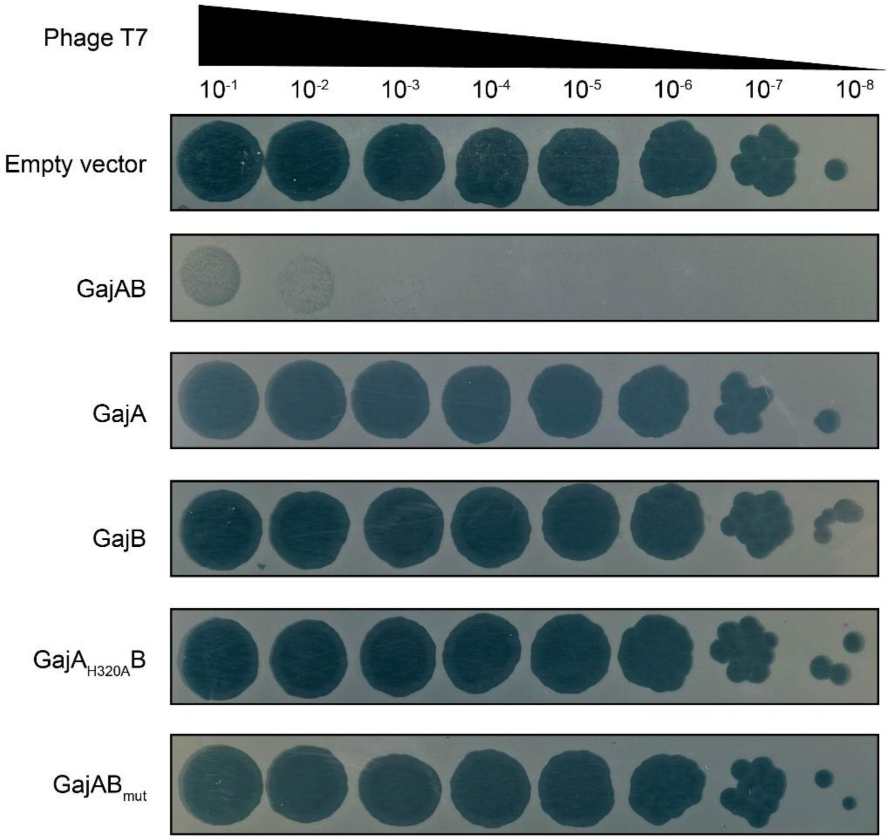

Supplementary Fig.2 Phage resistance assay of *E. coli* expressing various Gabija genes and an empty control vector. 10-fold dilutions of T7 phage solution infect *E. coli* harboring various Gabija genes and indicated mutant. Empty vector was used as a control. GajAB, wild type Gabija complex; $GajA_{H320A}B$, Gabija complex with H320A mutation in GajA; $GajAB_{mut}$, Gabija complex with D162A and E163A mutations in GajB; GajA, the GajA gene alone; GajB, the GajB gene alone.

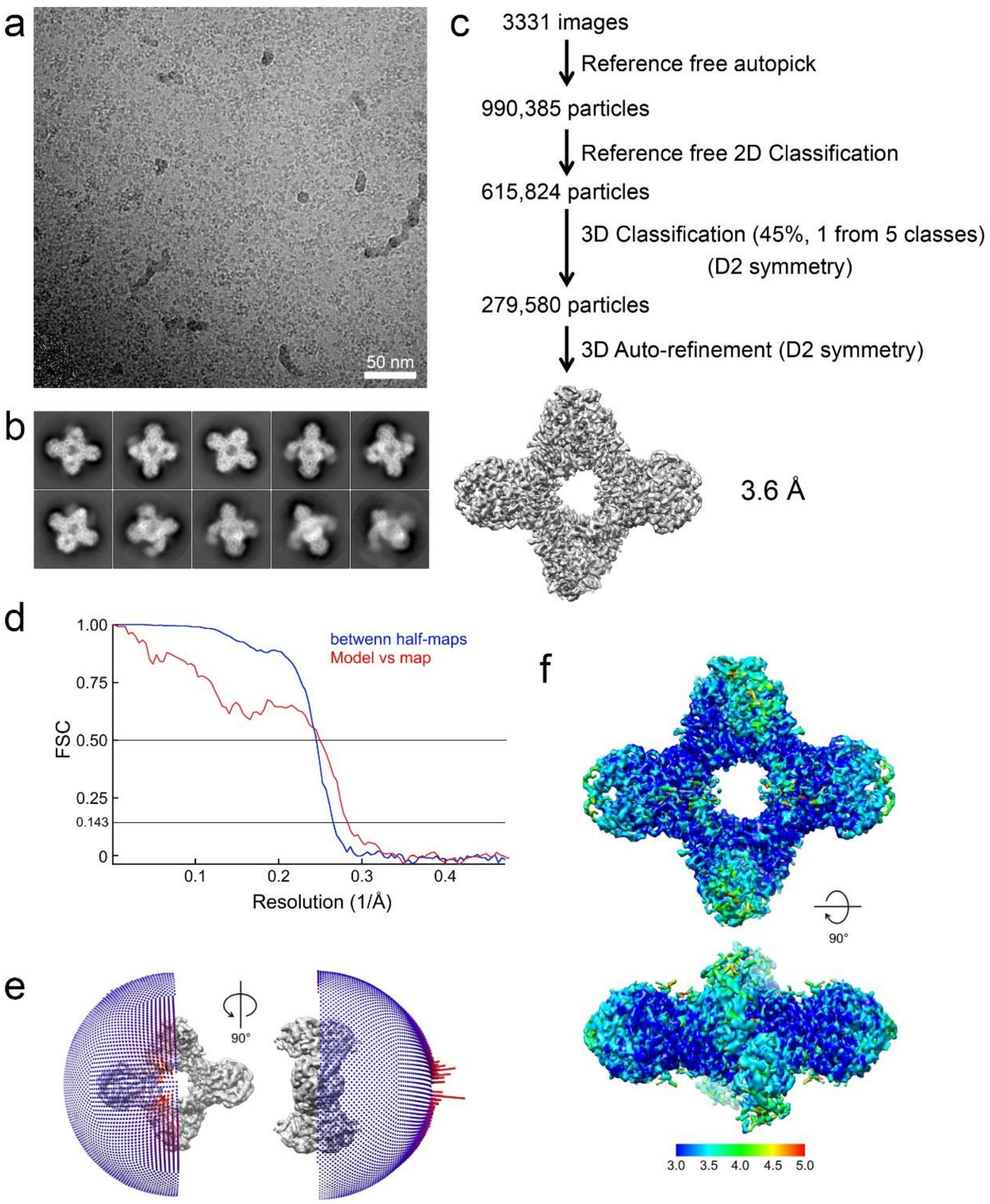

Supplementary Fig.3 Cryo-EM single-particle analysis of Gabija complex. a, Representative cryo-EM image of GajA-GajB complex. **b,** Representative reference-free 2D-class averages. **c,** Data-processing workflow of cryo-EM single-particle analysis. **d,** The gold standard Fourier shell correlation (FSC) curve for the final reconstruction. FSC curve between two half maps is indicated with resolutions at FSC=0.143. **e,** Angular distribution of particles in the final 3D auto-refinement. **f,** Local resolution map estimated by ResMap.

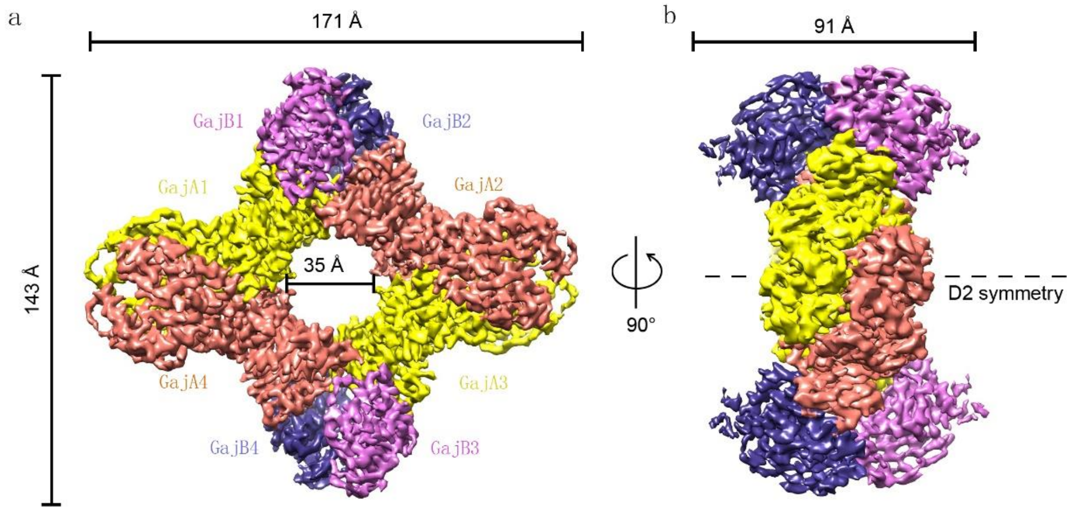

Supplementary Fig.4 Cryo-EM density map of Gabija complex shown in two different orientations. a, The top views of the Gabija complex. **b,** The side views of the Gabija complex. The D2 symmetry and the measurement of the octameric ring dimensions are marked. The four GajA are colored yellow and orange. The four GajB are colored blue and purple.

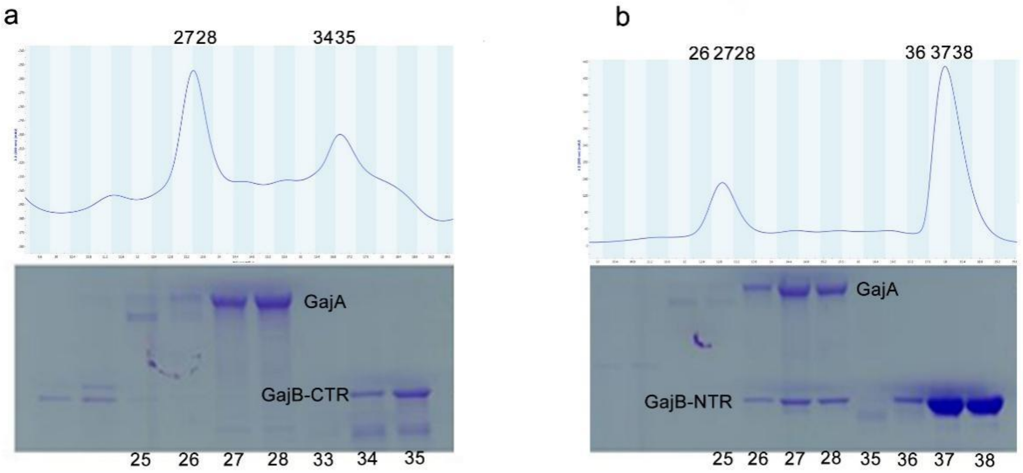

Supplementary Fig.5 Biochemical identification of interactions between GajA and GajB. (**a**, **b**) Size exclusion chromatography was used to analyze interaction between GajA and NTR region (residues 1-223) and the CTR region (residues 224-416) of GajB. Elution profile of Superdex 200 exclusion column is shown above and SDS-PAGE analysis of the peak fractions is shown below. Fraction numbers are labelled both in Elution profile of Superdex 200 and SDS-PAGE. GajA and GajB-CTR region migrate in separate peak in panel **a**, while GajA and GajB-NTR region elute in the same peak as shown in panel **b**.

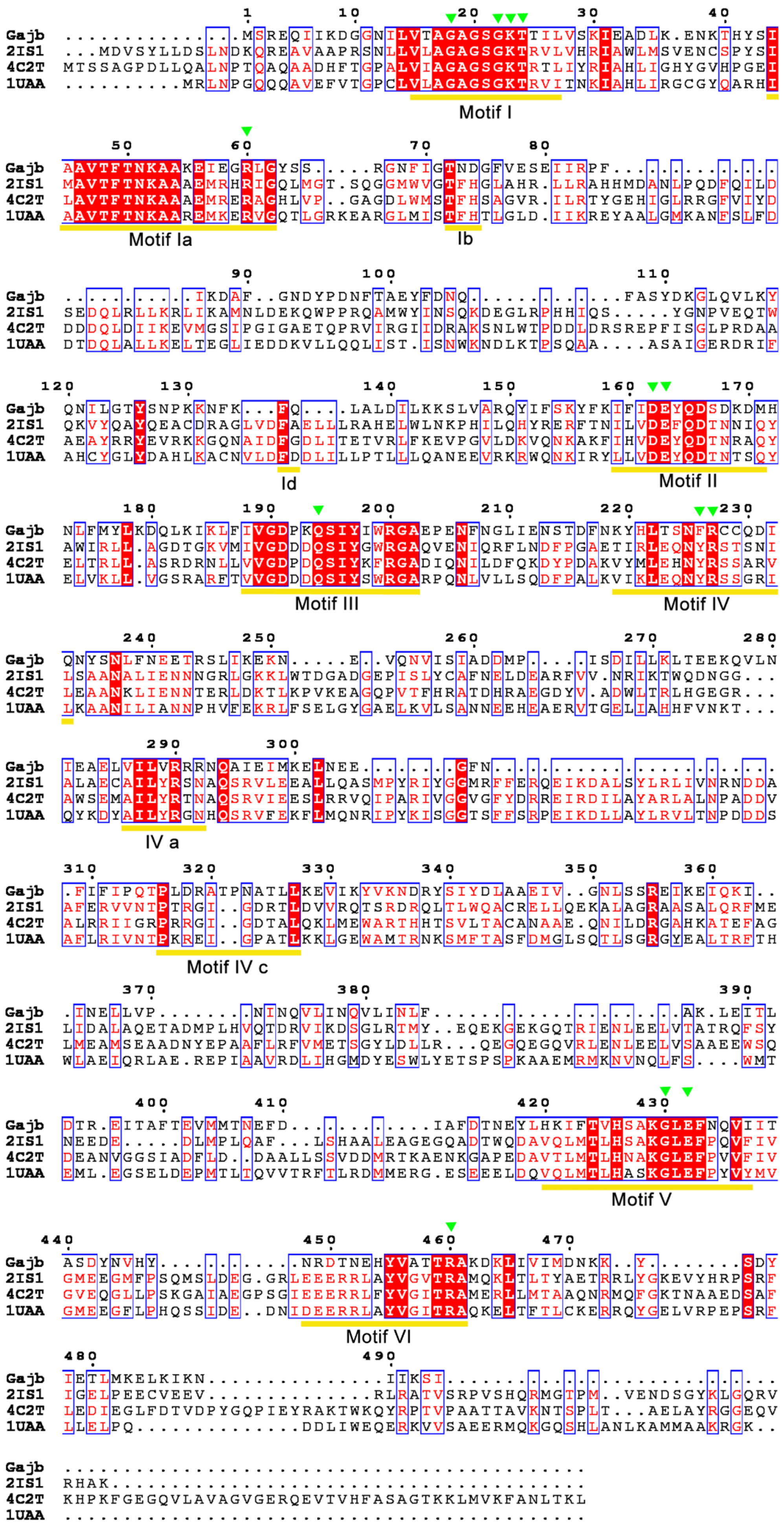
Gajb
2IS1
4C2T
1UAA
Motif I
Motif Ia
Ib
Id
Motif II
Motif III
Motif IV
IV a
Motif IV c
Motif V
Motif VI

Supplementary Fig.6 Sequence conservation analysis. Sequence alignment among SF1 helicase from GajB, 2IS1, 4C2T, 1UAA. The absolutely conserved residues are boxed in red, and highly conserved ones are colored in red and in unfilled boxes. Residues involved in the binding of ATP is marked with green triangle; The Conserved Sequence Motifs are highlight by yellow underline.

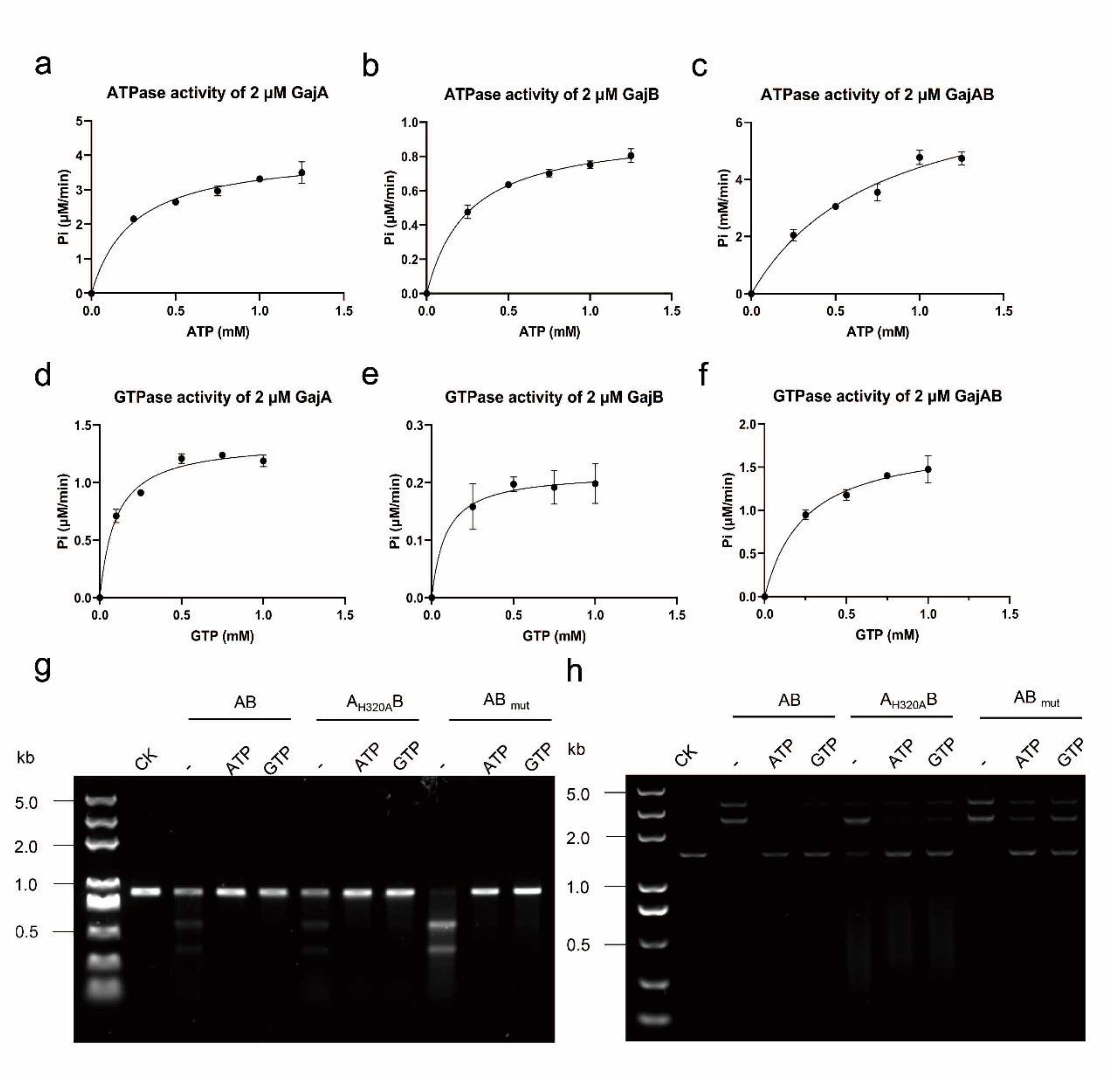

Supplementary Fig.7 Investigation of Gabija complex ATPase domains by Michaelis–Menten kinetics and site-specific mutagenesis. (**a**,**b**,**c**) Positive synergistic effect of ATP hydrolysis capacity of Gabija complex is illustrated by Michaelis–Menten kinetics of hydrolysis activities of GajA, GajB and Gabija complex. Nonlinear regression to the Michaelis-Menten equation and statistical analysis were performed using GraphPad Prism. Each data point is the average of three independent experiments and SD error bars is indicated. (**d**,**e**,**f**) GTP hydrolysis activities of GajA, GajB and Gabija complex is measured by Michaelis–Menten kinetics. (**g**,**h**) The effect of mutations in GajA or GajB ATPase active site on the ATP/GTP inhibition of Gabija complex activity. The DNA substrate is linear λ955 and the concentration of ATP/GTP is 0.5mM in panel **g**, while the DNA substrate is pUC19-15 plasmid and the concentration of ATP/GTP is 2mM in panel **h**. CK, control reaction without Gabija complex. AB, wild type Gabija complex; $A_{H320A}B$, Gabija complex with H320A mutation in GajA; AB_{mut}, Gabija complex with D162A and E163A mutations in GajB.

D162A and E163A mutations in GajB but not H320A mutation in GajA mutation partially relieved the inhibition of ATP/GTP on Gabija complex nuclease activity in the presence of pUC19-15 plasmid substrate. Lanes labeled with dashes indicate no nucleotide addition.

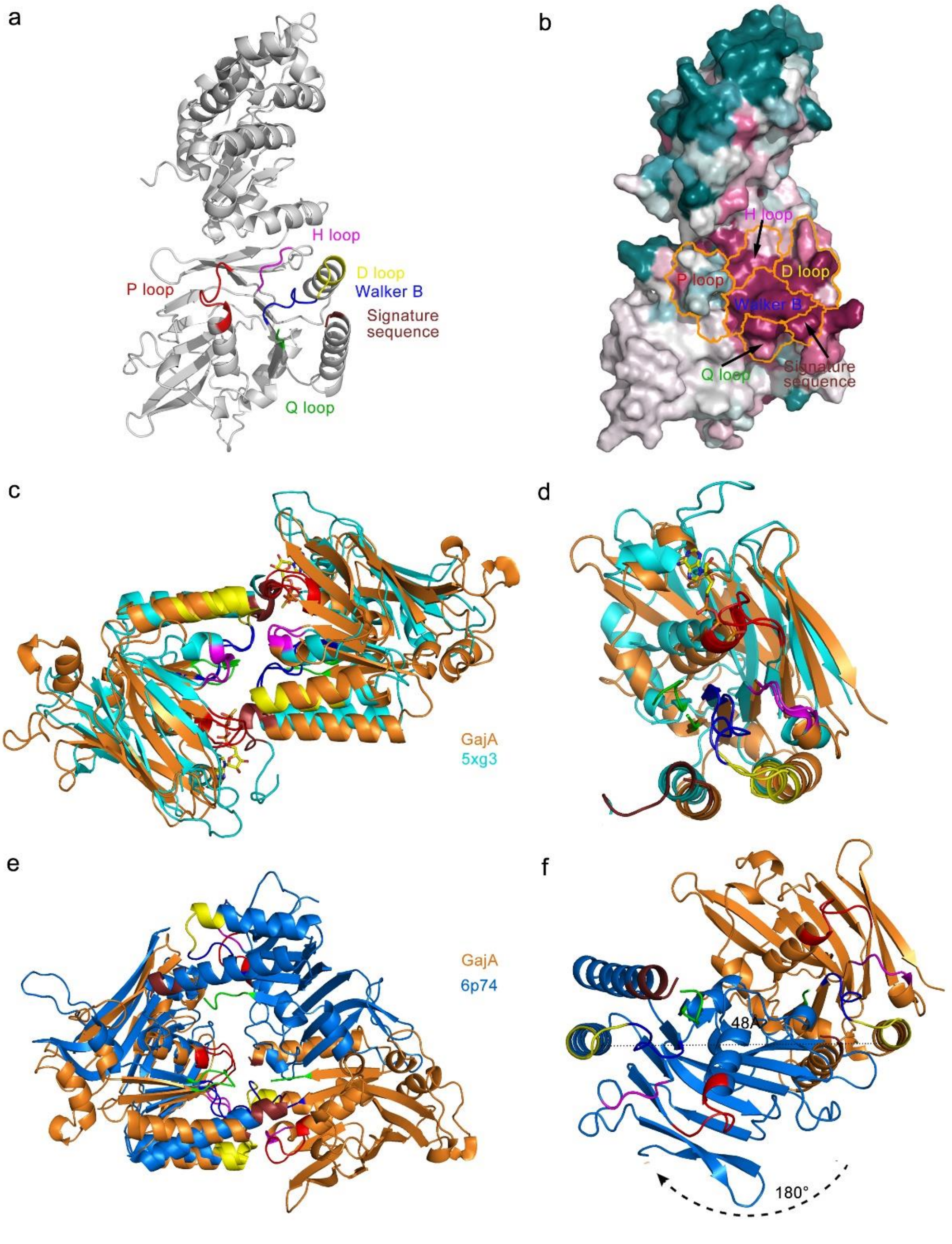

Supplementary Fig.8 GajA ATPase domains have a conserved active site and adopt a productive conformation. a, Mapping of the catalytic motifs of ABC ATPases onto the GajA structure and colored as follows: P loop, red; Q loop, green; Walker B, blue; D loop, yellow; ABC signature sequence, ruby; H loop, purple. **b**, Surface conservation of Catalytic motifs of ABC ATPases in the GajA structure by ConSurf server. P loop fail to give reasonable results due to insufficient data. **c**, Structural superposition of the ATPase domain dimers of GajA (orange) and

chromosome partition protein SMC (cyan, PDB:5xg3). The left Aligned domains in each structure is Subunit 1, The right one is Subunit 2. **d**, Close-up view of the aligned ATPase domains (Subunit 2) from dimer superposition in **c**. **e**, Structural superposition of the ATPase domain dimers of GajA (orange) and OLD nuclease (marine, PDB:6p74). The left Aligned domains in each structure is Subunit 1, The right one is Subunit 2. **f**, Close-up view of non-aligned aligned ATPase domains (Subunit 2) from dimer superposition in **e**. Dashed black arrow and Dashed black line indicates rotation and distance during the conformation change of ATPase domains of GajA relative to its counterpart of OLD nuclease.

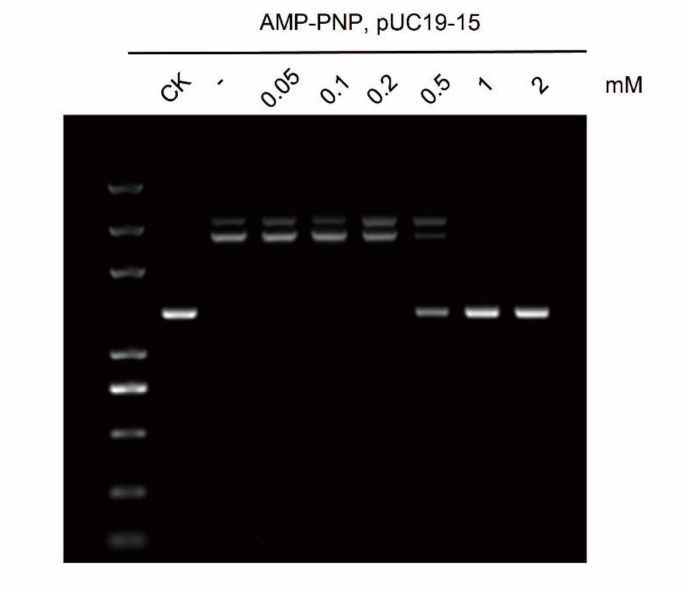

Supplementary Fig.9 Effect of AMP-PNP on nuclease activity of Gabija complex in the presence of pUC19-15 substrate.

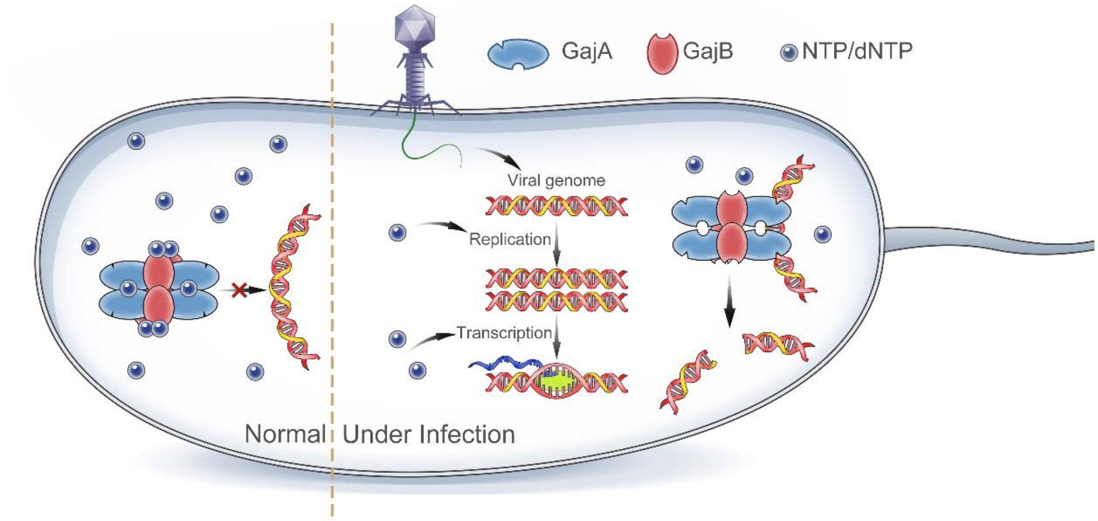

Supplementary Fig.10 Model of Gabija anti-phage defense mechanism. Under normal conditions, the Gabija complex nuclease activity is fully inhibited by nucleotides at physiological concentration in bacteria, Both the ATPase domain of GajA and GajB bind NTP and dNTP to allosterically regulate the TOPRIM domain. Under phage invasion, the high-intensity DNA replication and transcription markedly reduced the concentration of nucleotides in the cell. As the NTP and dNTP concentrations decrease to a certain degree, the loss of nucleotide binding of ATPase domain activates the nuclease activity of TOPRIM domain, and Gabija complex begin to cleave circular and linear DNA in order. When the ATP concentration is decreased to (0.5mM~2mM ATP) from 3mM physiological concentration. the cleavage activity of the Gabija complex on supercoiled circular DNA is activated. Later, the ATP concentration is decreased to below 0.5mM, the cleavage activity of the Gabija complex on linear DNA is activated. Thus, the Gabija complex cuts the phage DNA, stopping the infection due to phage replication failure, and may also cleave bacterial genomic DNA for abortive infection.

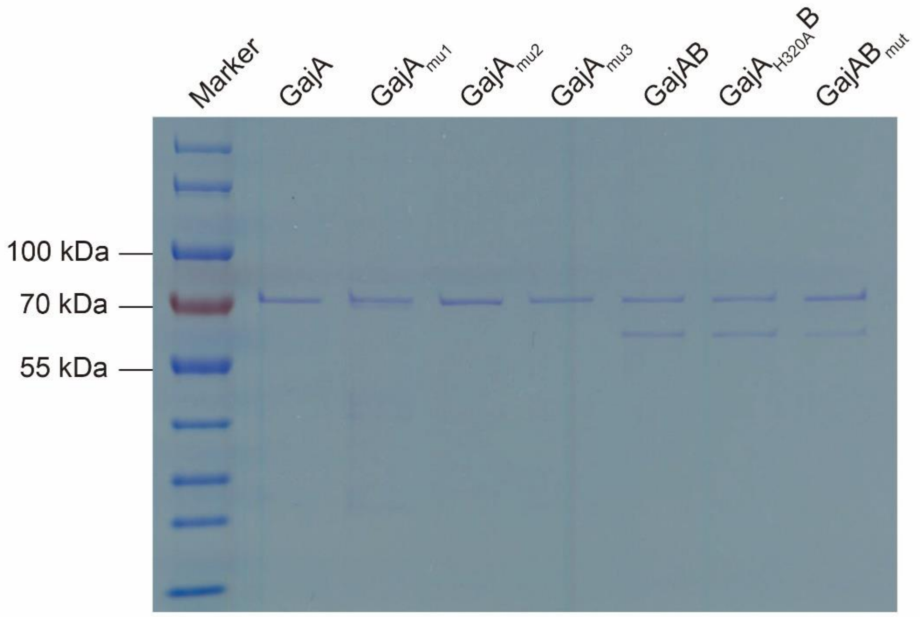

Supplementary Fig.11 SDS-PAGE characterization of purified wild-type GajA and indicated GajA mutants. GajA, wild type GajA; GajA$_{mut1}$, GajA mutant with (K436A, K438A, K439A, K441A, K442A); GajA$_{mut2}$, GajA mutant with (R481A, R485A, K487A, K491A); GajA$_{mut3}$, GajA mutant with (K474A, K476A, K477A, K478A, K479A); GajAB, wild type Gabija complex; GajA$_{H320}$AB, Gabija complex with H320A mutation in GajA; GajAB$_{mut}$, Gabija complex with D162A and E163A mutations in GajB.

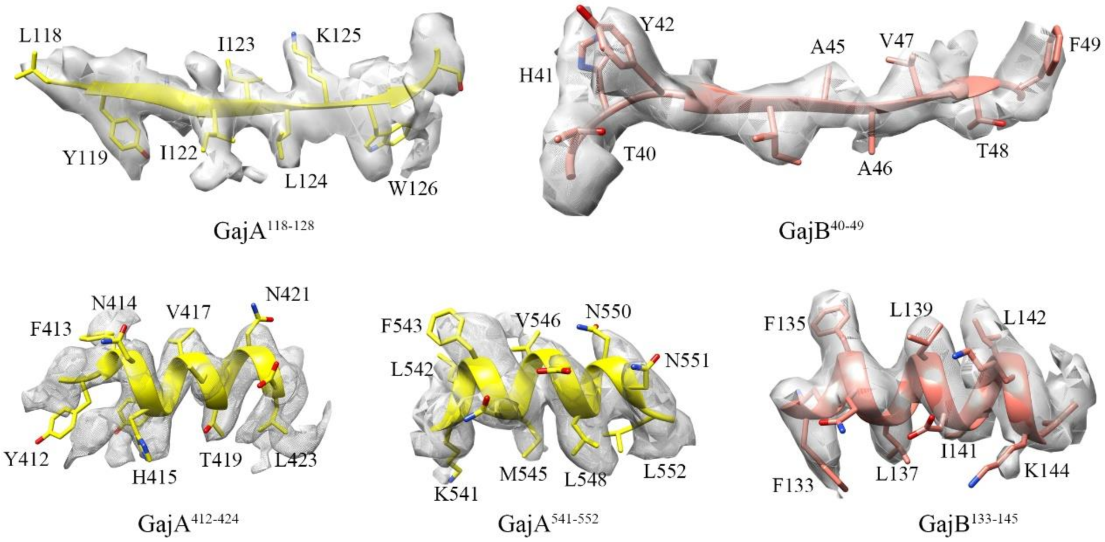

Supplementary Fig.12 GajA and GajB map to model fit for designated regions.

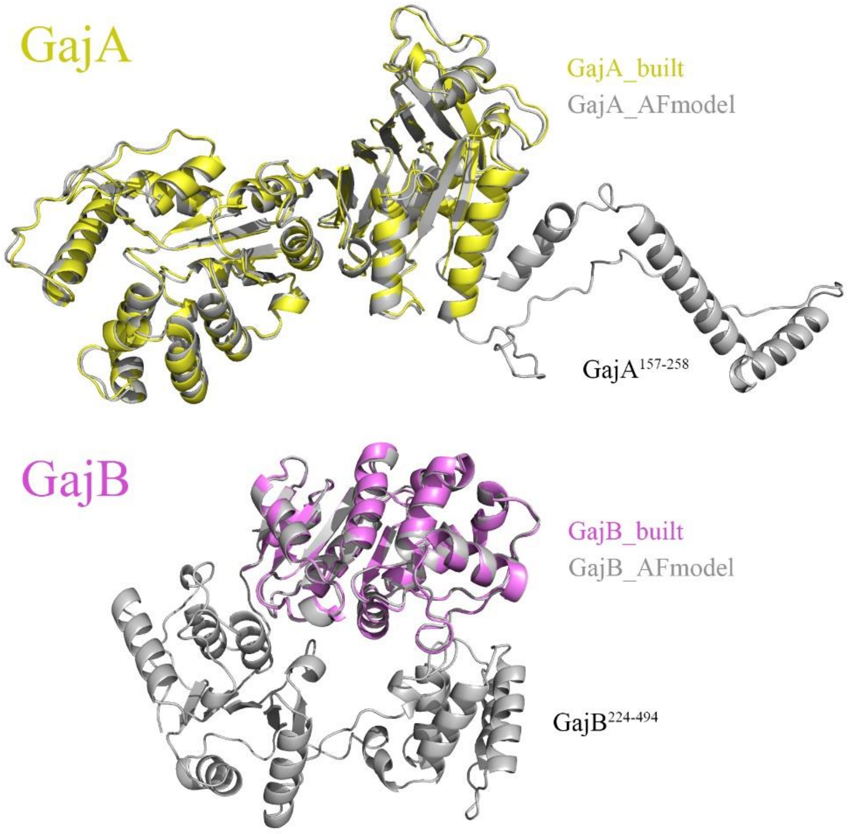

Supplementary Fig.13 Comparison of Alphafold 2 predicted model and final built model in GajA and GajB.

Supplementary Table 1. Cryo-EM data collection and validation statistics

Gabija complex (EMD-35977, PDB 8J4T)	
**Data collection and processing**	
Microscope	Talos Arctica
Detector	K2 Summit
Magnification (nominal/calibrated)	130,000
Voltage (kV)	200
Electron exposure ($e^-/Å^2$)	60
Defocus rang (µm)	1.0-3.0
Pixel size (Å)	1.0
Symmetry imposed	D2
Initial particle images (no.)	990,385
Final particle images (no.)	279580
Map resolution (Å)	3.6
FSC threshold	0.143
Map resolution range (Å)	3.0-7.5
Map sharpening *B* factor ($Å^2$)	-201.93
**Model refinement and validation**	
Initial model used	Alphafold 2 predicted model
Model resolution (Å)	3.6
Model resolution range (Å)	∞-3.6
Model composition	
Non-hydrogen atoms	45756
Protein residues	2796
Ligands	-
B factors ($Å^2$)	
Protein	73.68
Ligand	-
R.m.s. deviations	
Bond lengths (Å)	0.011
Bond angles (°)	1.14
Validation	
MolProbity score	1.91
Clashscore	7.17
Rotamer outliers (%)	0
Ramachandran plot	
Favored (%)	91.16
Allowed (%)	8.84
Disallowed (%)	0

Supplementary Table 2. Primers used in this study.

Primer name		5′-3′ sequence
GajA-52b-mu1	Forward	GCAGGTGCAGCGGGCGTTTACGAACTGCTGGGCCTGA
	Reverse	GGCACTTGCCAGGTCATTATCGGTCTTAATAATATGGG
GajA-52b-mu2	Forward	TGAATGAAGCCAAAGCAGAAATTTTTGCGCAGTAC
	Reverse	GCAAAAATTTCTGCTTTGGCTTCATTCAGAGCCTC
GajA-52b-mu3	Forward	GCGGCCGCAGAGCGTCTGAATGAACGCAAAAAAGAAATT
	Reverse	GGCACCTGCAATATCTTCCGGGATATCAATGGTAATTTC
GajA-H320A-52b	Forward	CCTGAGTACCGCTAGTCCGGAACTGCTGTATGAAATG
	Reverse	CCGGACTAGCGGTACTCAGGAAGAAATATTTATAGGTA
GajB_CTR(224-494aa)	Forward	AGCAATTTTCGCTGCTGTCAGGATATTCAGAAT
	Reverse	ATGGCTGCCGCGCGGCACCAGGCCGCTGCT
GajB_NTR(1-223aa)	Forward	TAACTCGAGCACCACCACCACCACCACTGA
	Reverse	GGTCAGATGGTACTTATTAAAATCGGTGCTATTTT
GajB-D162AE163A	Forward	CAAGATCTTCATCGCCGCATACCAGGATAGCGATAAAGATATG
	Reverse	ATCCTGGTATGCGGCGATGAAGATCTTGAAATATTTGCTAAA
GajAB-A-H320A-PQE82L	Forward	TTCTTATCCACTGCCTCTCCTGAACTTCTTTATGAAATGGATAATAC
	Reverse	ATAAAGAAGTTCAGGAGAGGCAGTGGATAAGAAAAAATATTTATATGTAG
GajAB-B-D167A-E168A-PQE82L	Forward	AAGATATTTATAGCCGCGTACCAAGATTCGGATAAGGATATGCATAATT
	Reverse	ATCCGAATCTTGGTACGCGGCTATAAATATCTTGAAGTATTTTGAAAAT
λ955	Forward	GCAGATTATTATGGGCCGCCACGAC
	Reverse	AACTGCCCTCCAAATCCGCTGC
pUC19-15	Forward	TCGATTTTTGTGATGCTCGTCAGGGG
	Reverse	AAAAATAGGCGTATCACGAGGCCCT
