# Supplementary material for: Structural and biochemical insights into the mechanism of the Gabija bacterial immunity system
Source: Nat Commun. 2024 Jan 29;15:836. doi: 10.1038/s41467-024-45173-7 (PMC10822852; doi:10.1038/s41467-024-45173-7)

Source data

Fig.4 b (left)-c (right)

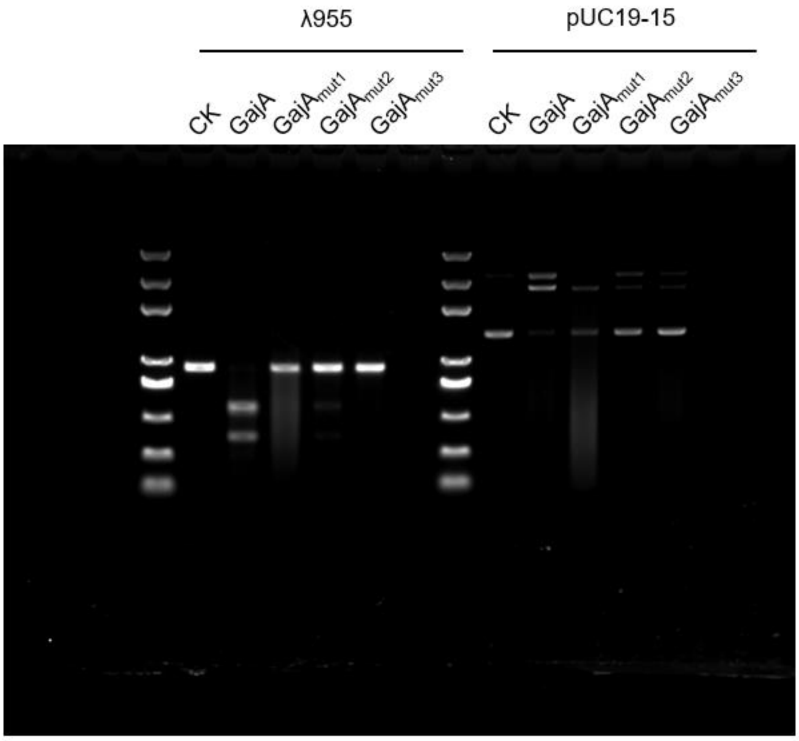

Fig.5 a

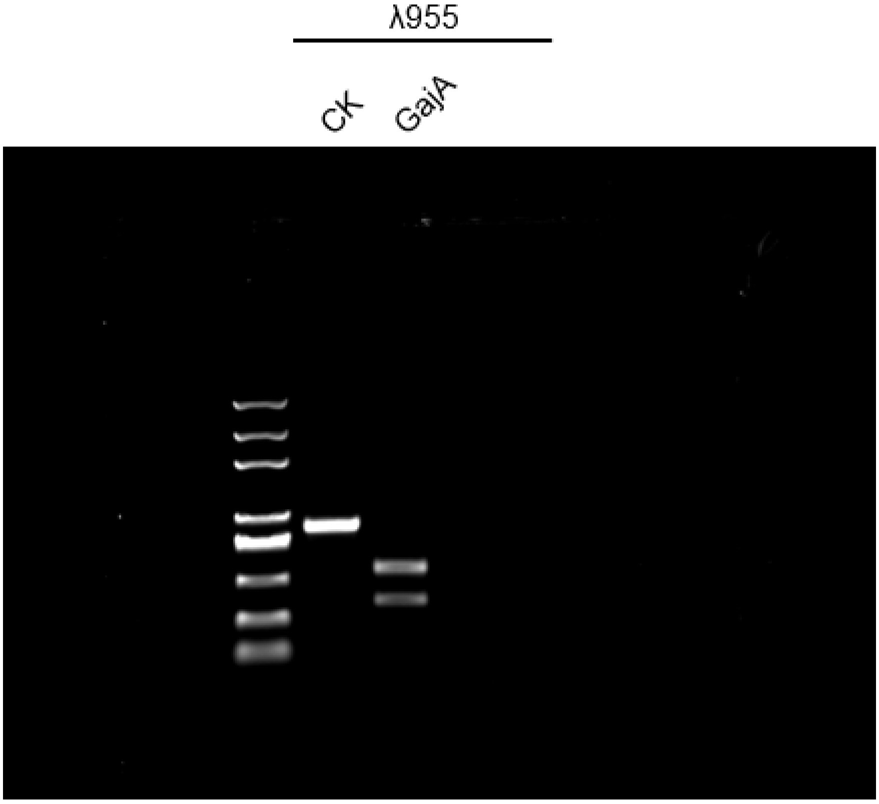

Fig.5 b

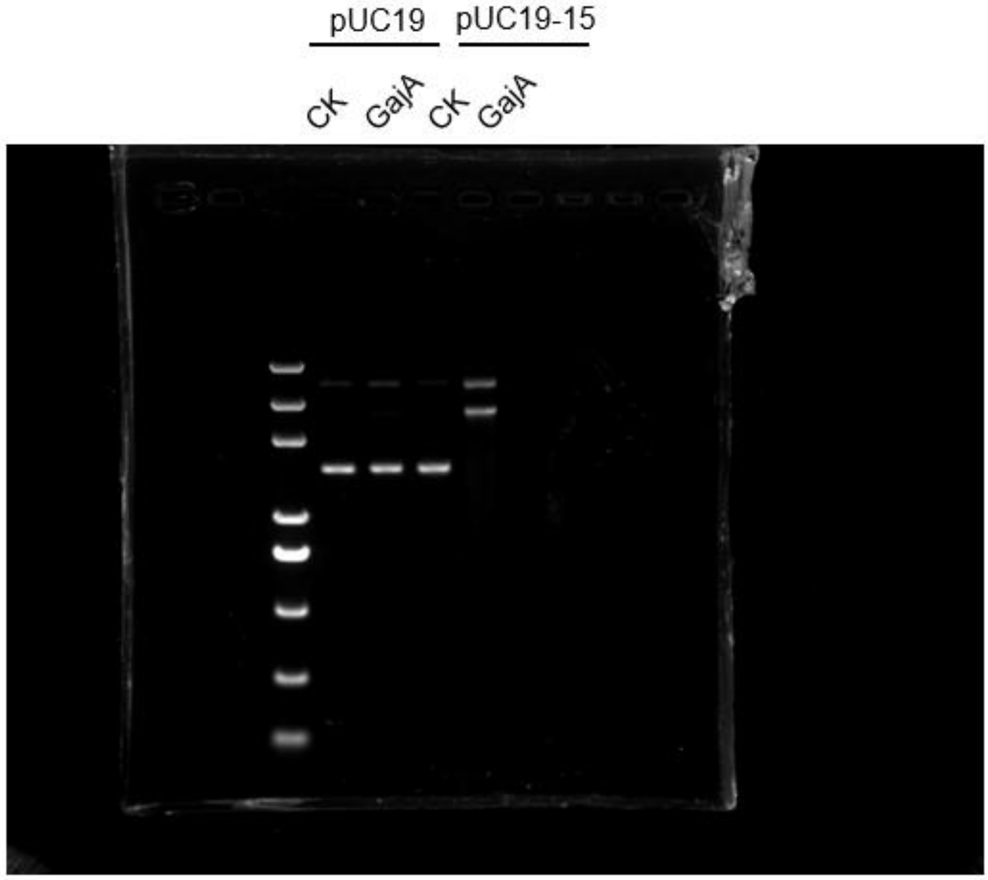

Fig.5 c

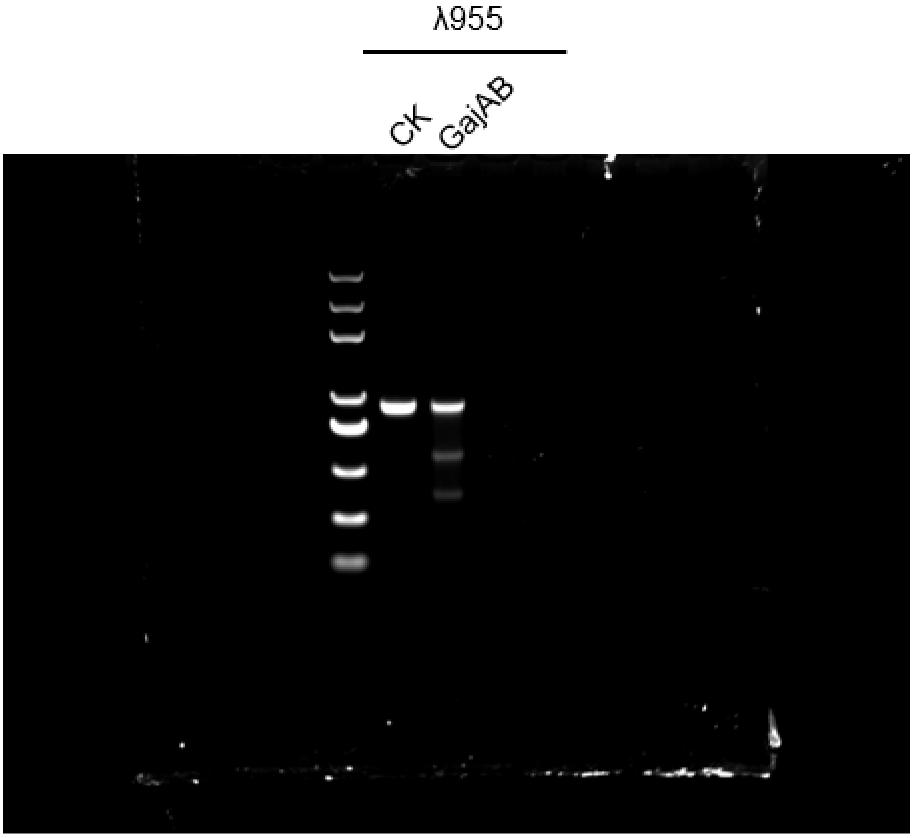

Fig.5 d

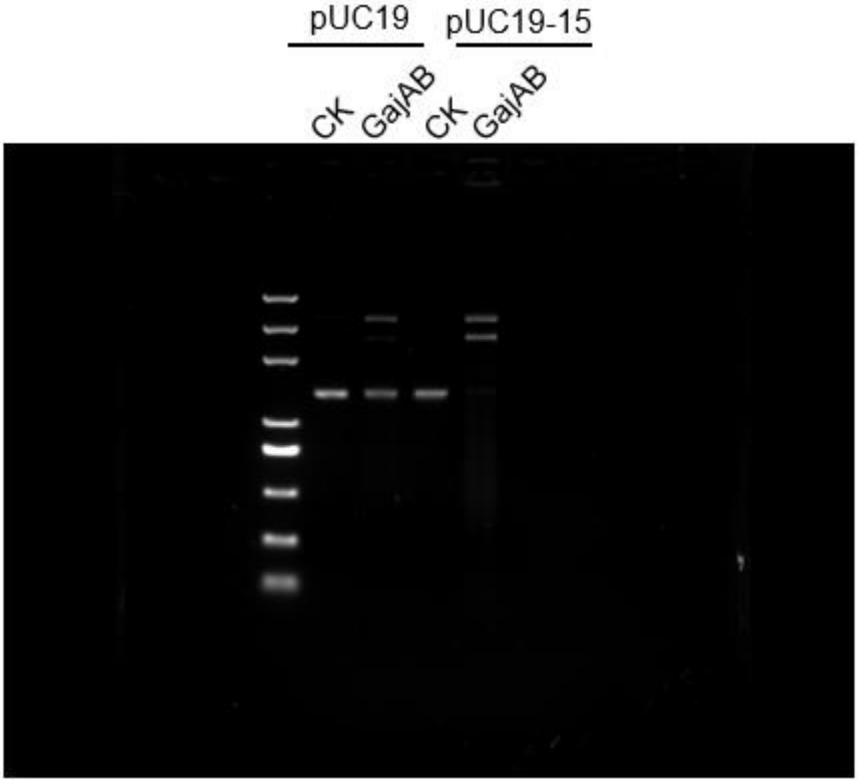

Fig.6 a

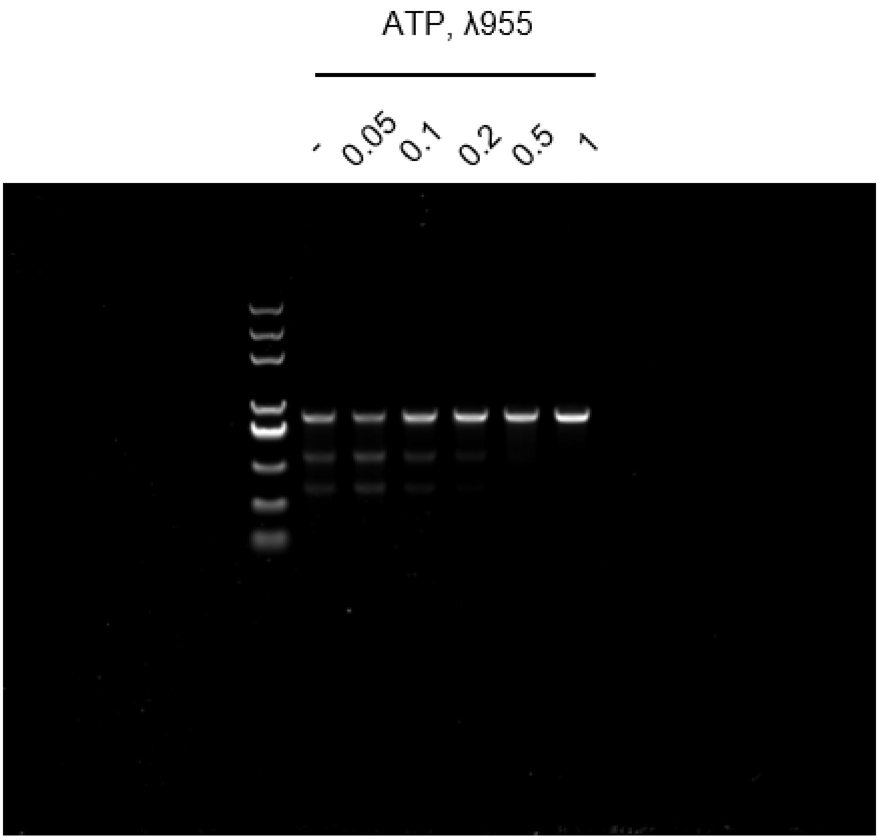

Fig.6 b

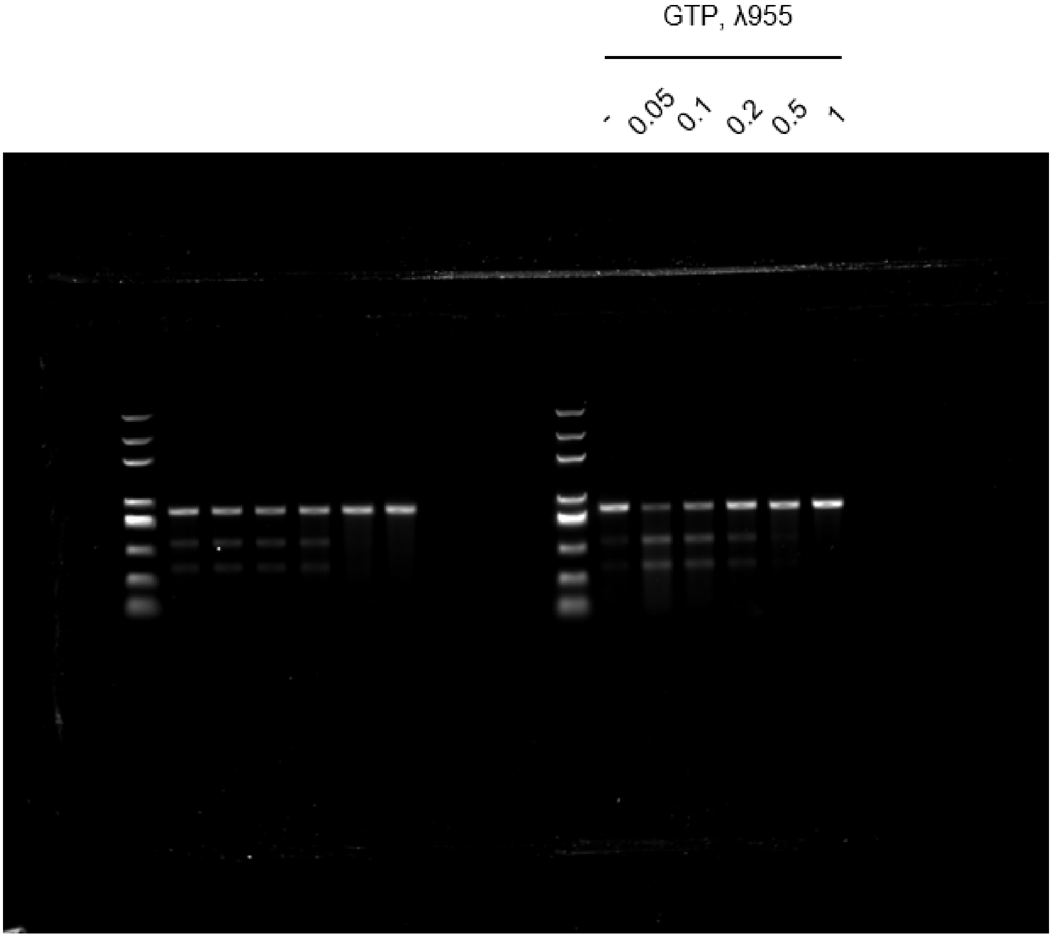

Fig.6 c (left)-d (right)

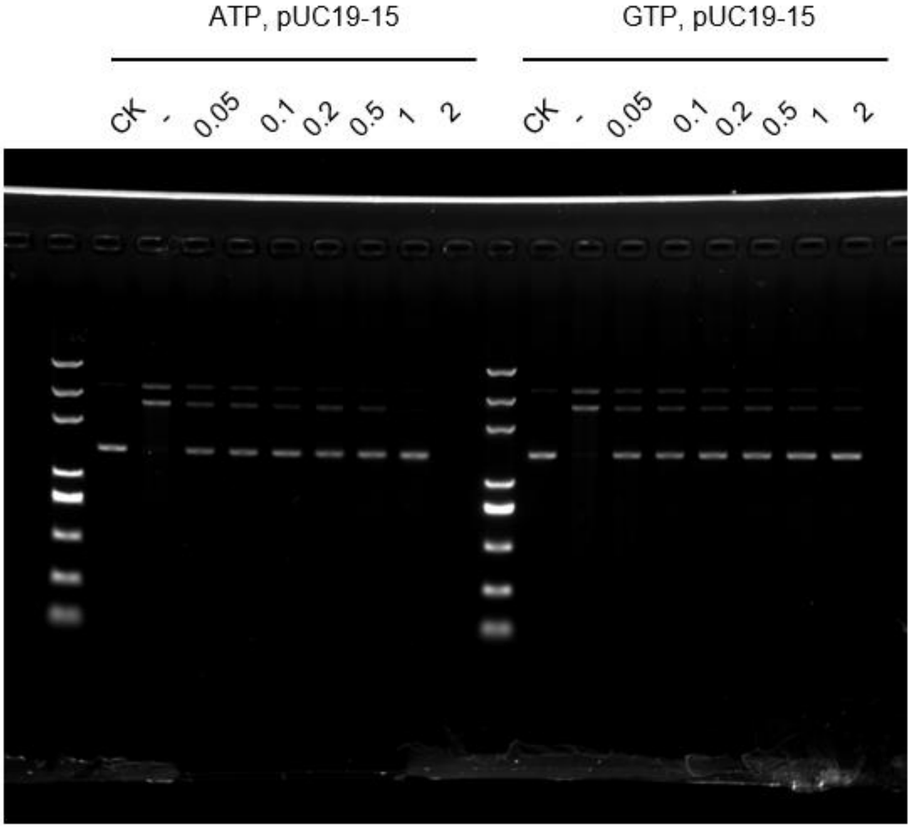

Fig.6 e

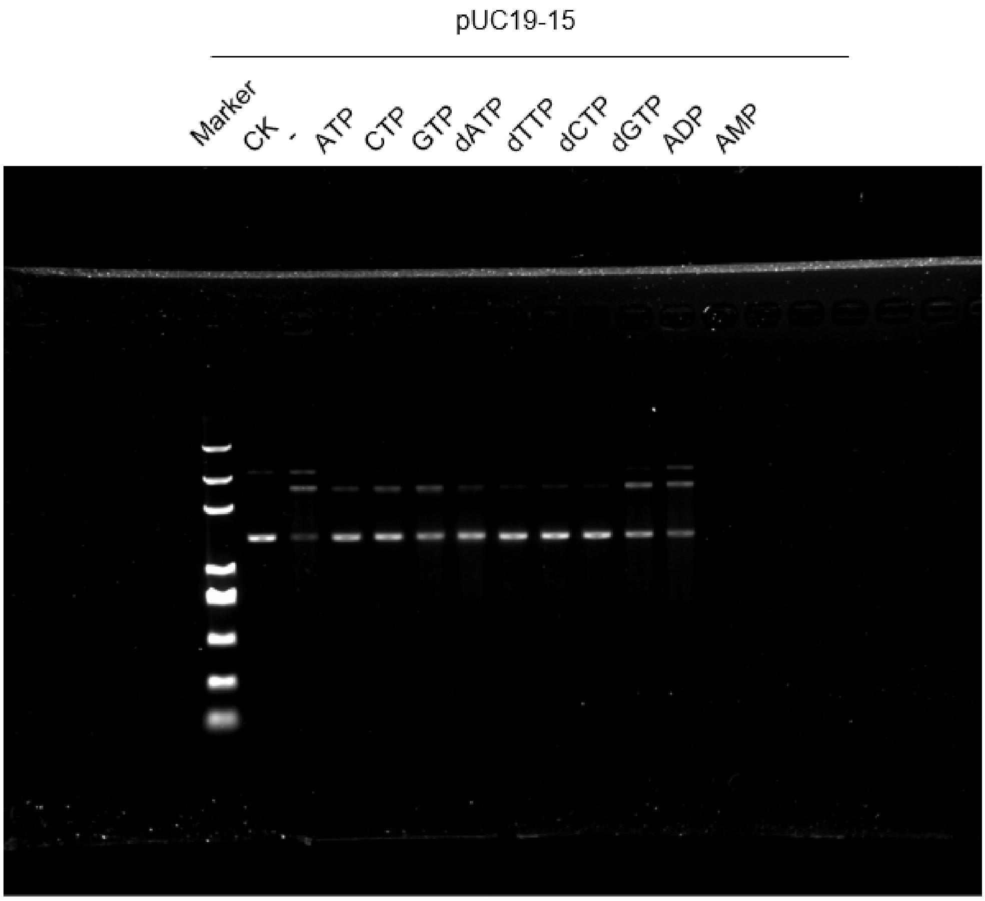

Fig.6 f

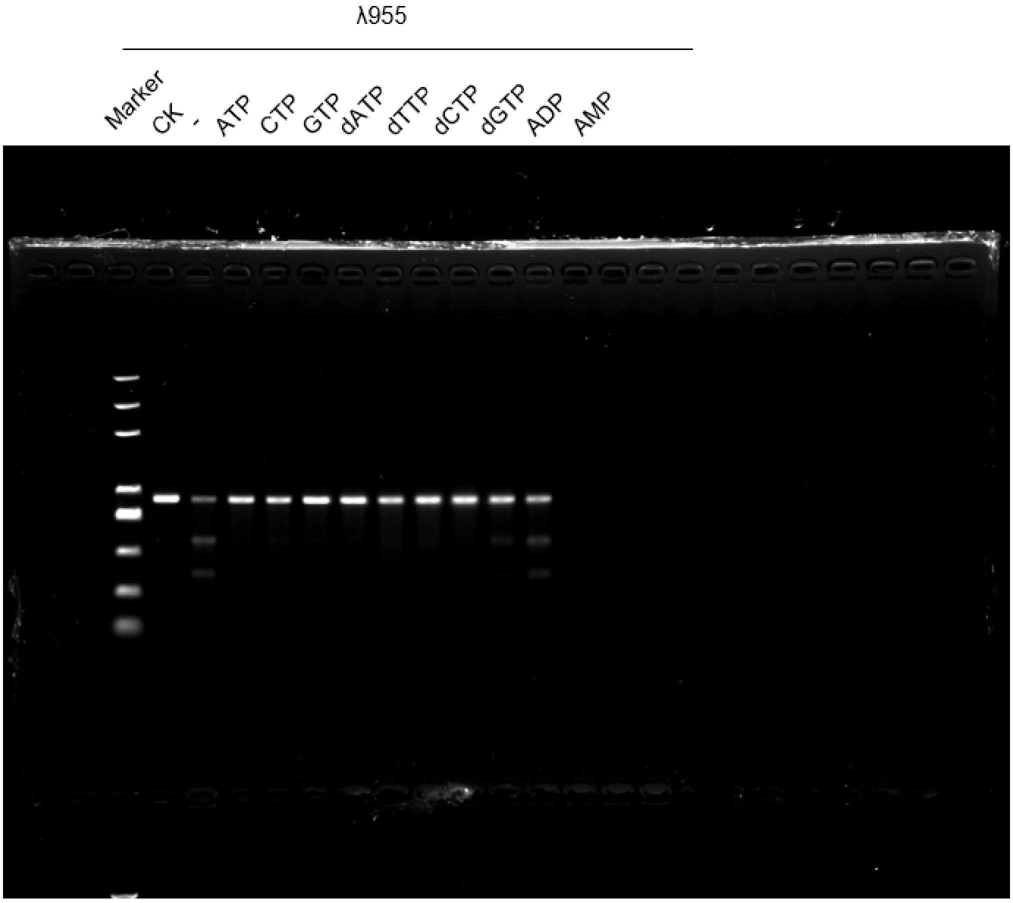

Supplementary Fig.1 a

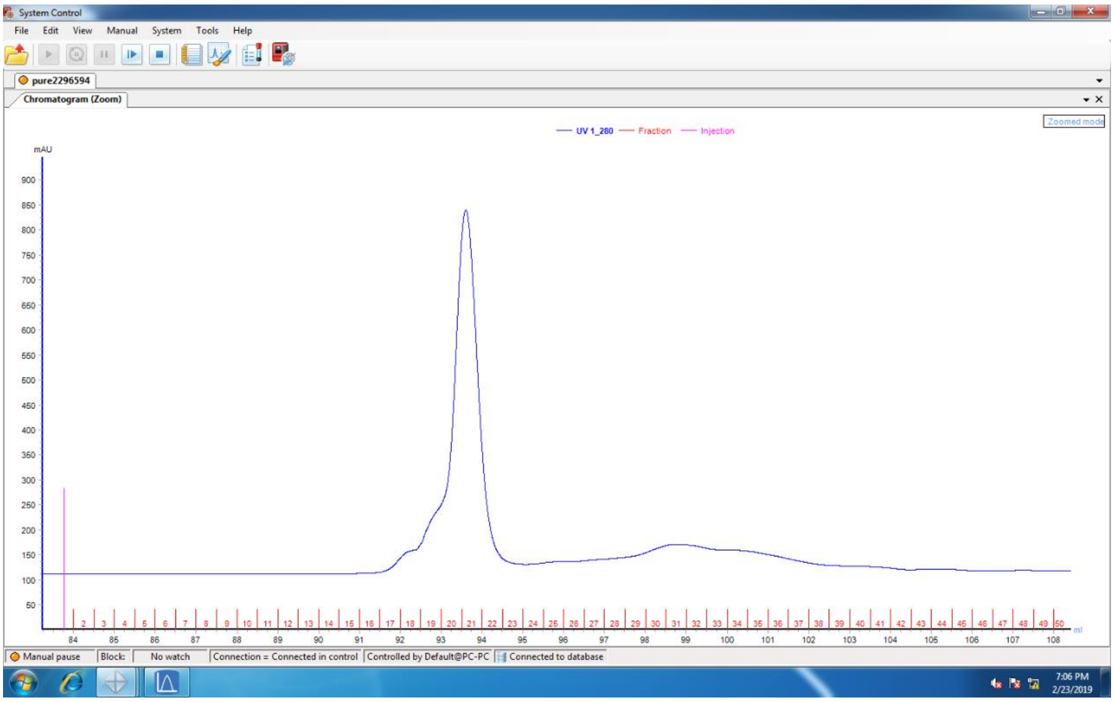

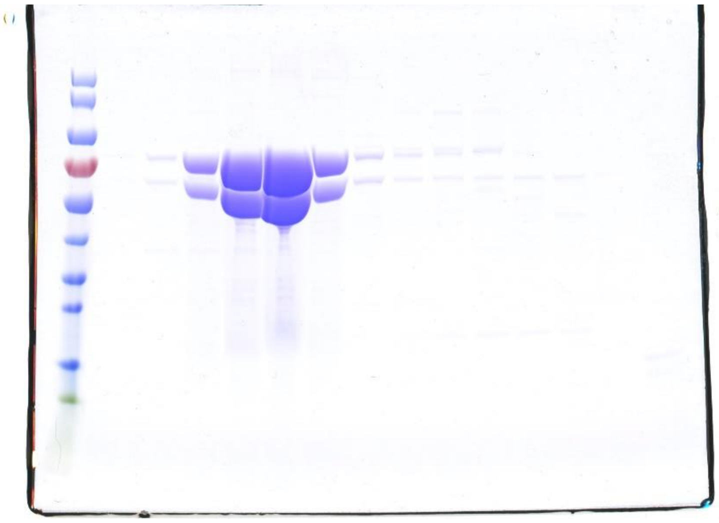

Supplementary Fig.2

Empty vector

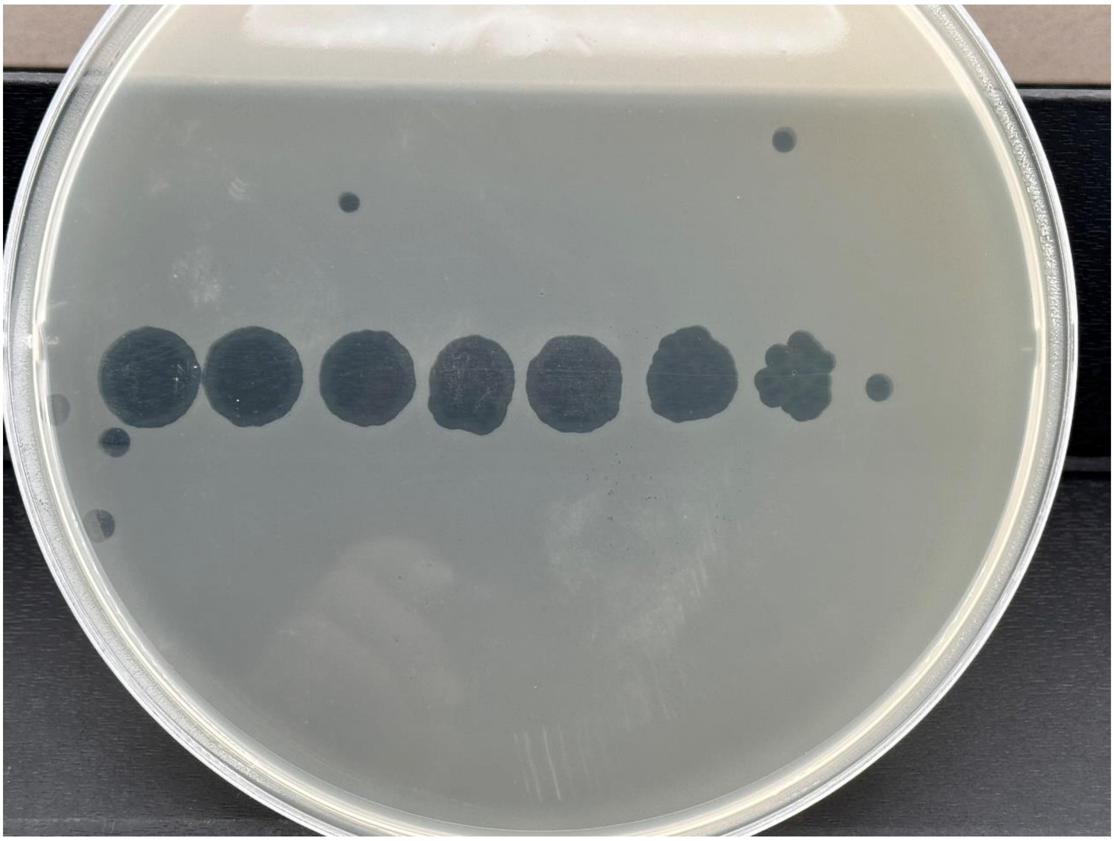

Supplementary Fig.2

GajAB

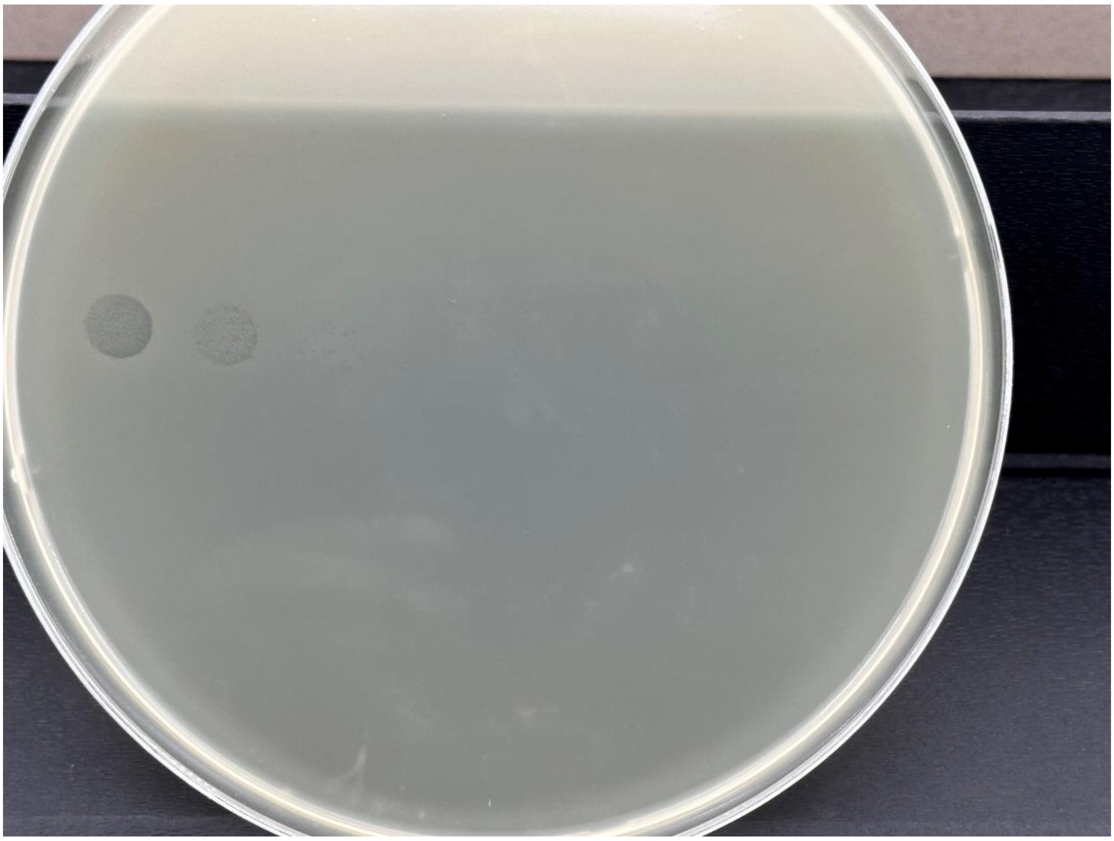

Supplementary Fig.2

GajA

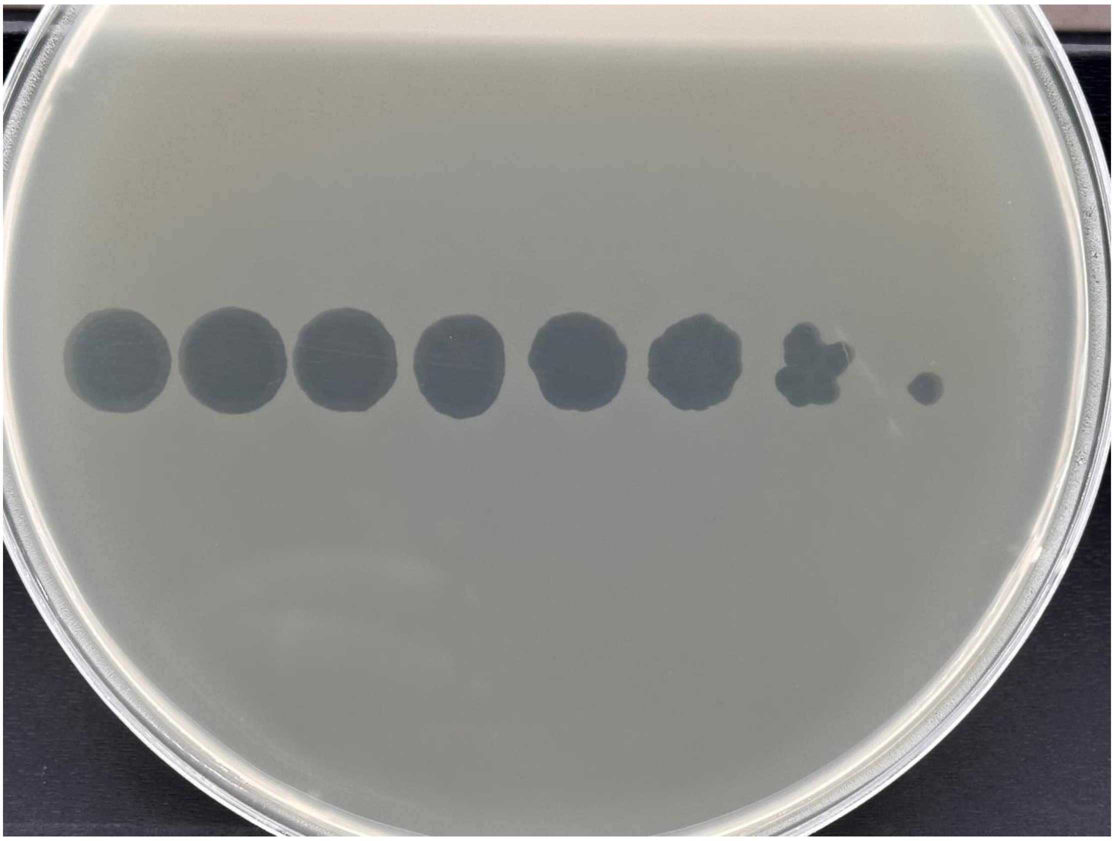

Supplementary Fig.2

GajB

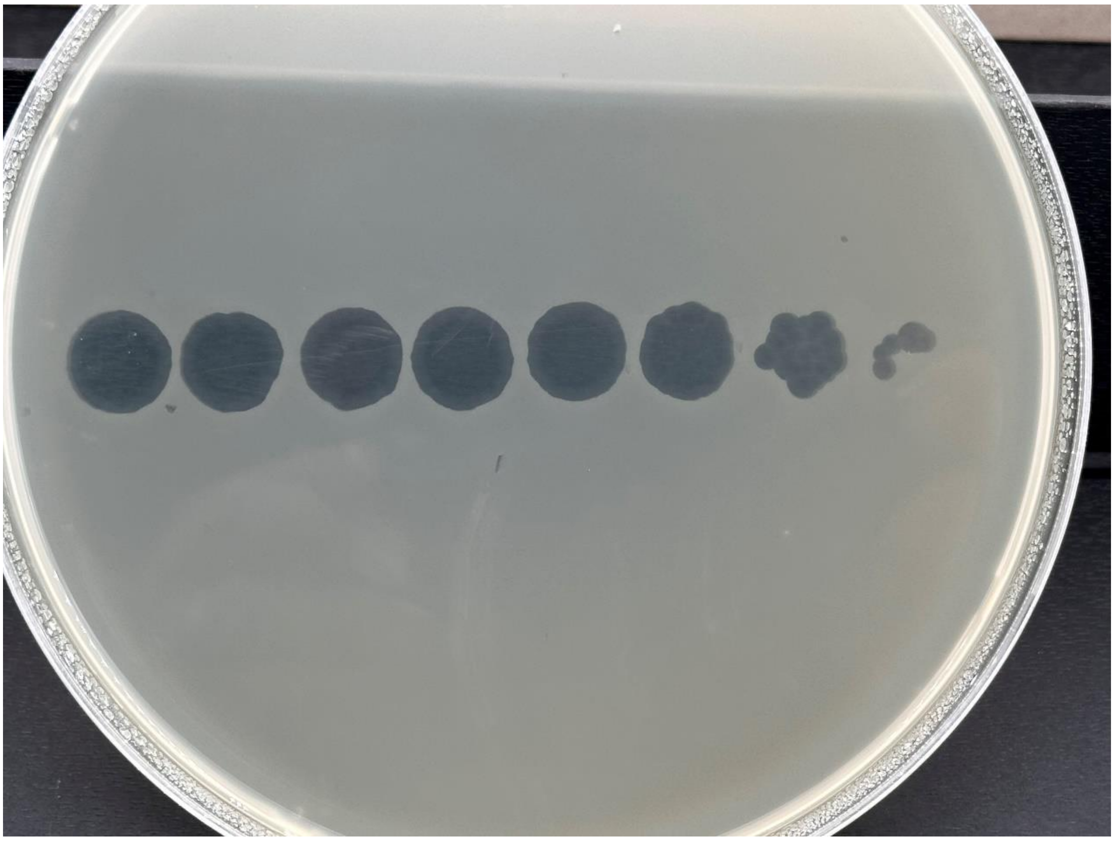

Supplementary Fig.2

GajA$_{H320A}$B

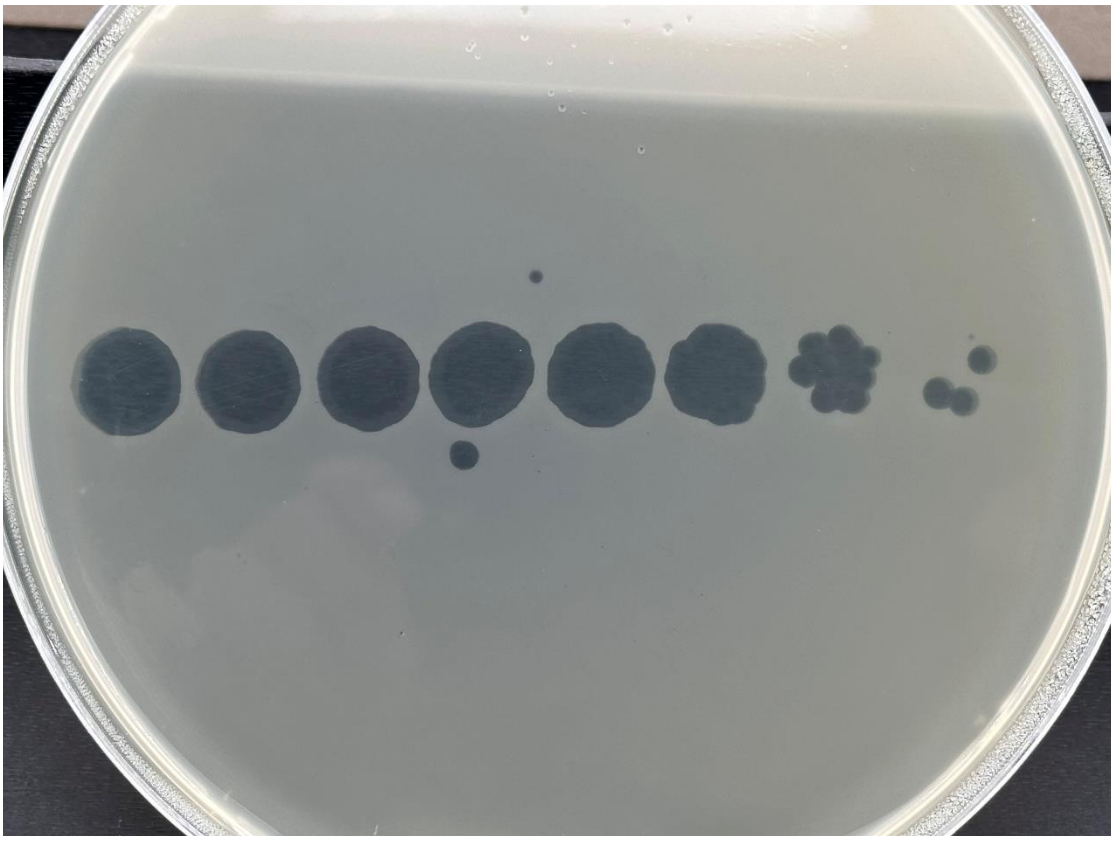

Supplementary Fig.2

GajAB$_{mut}$

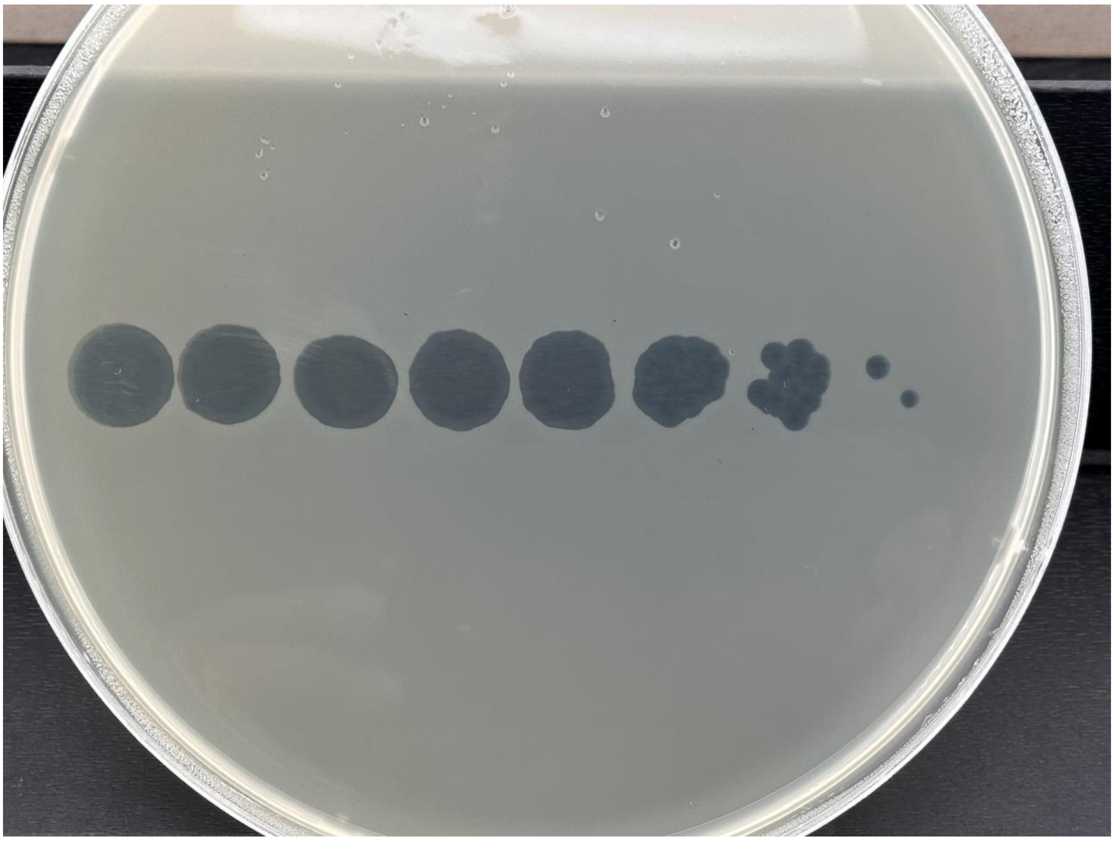

Supplementary Fig.5 a

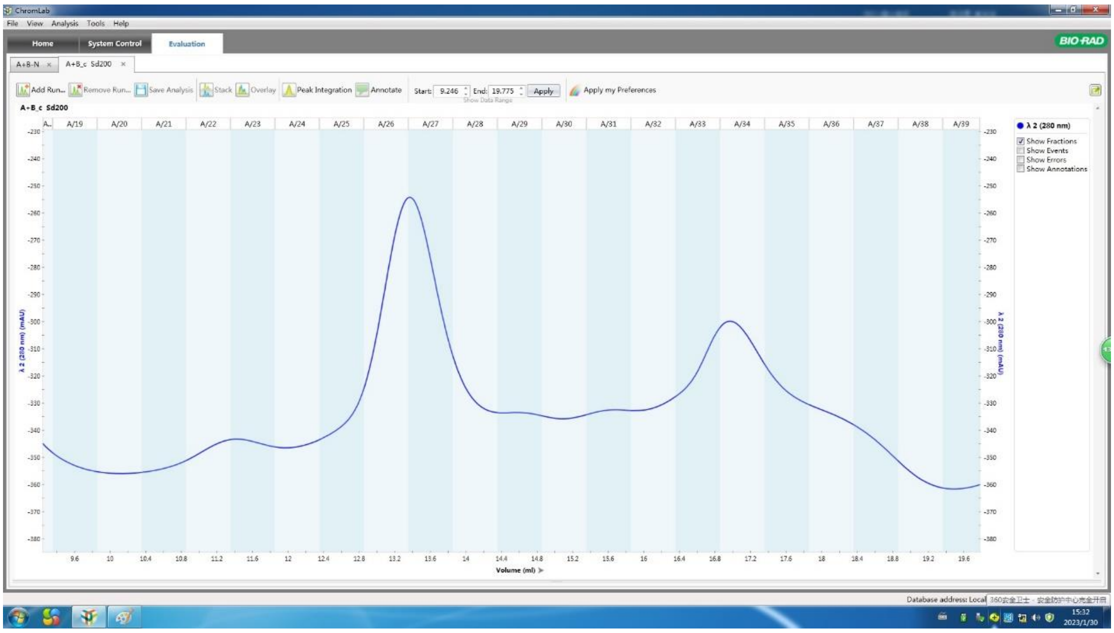

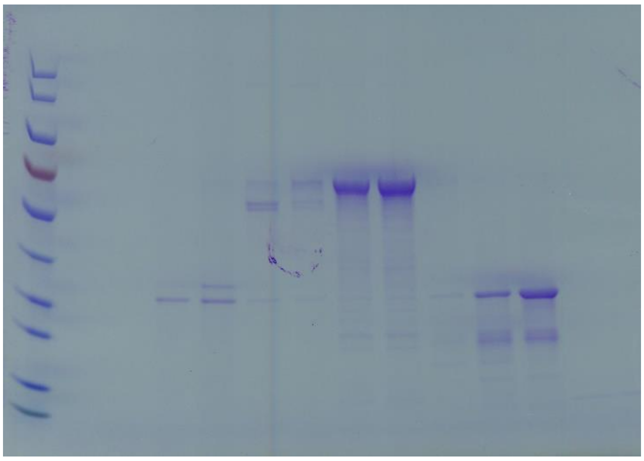

Supplementary Fig.5 b

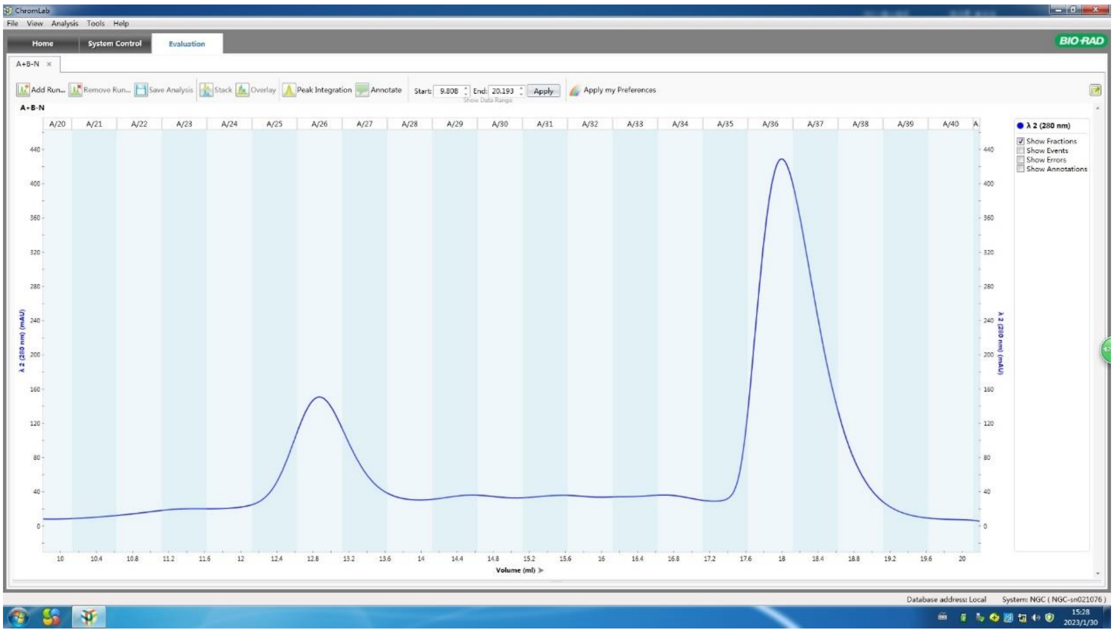

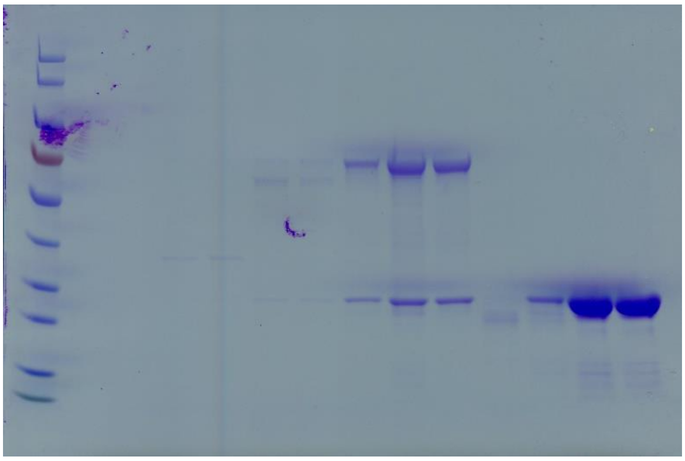

Supplementary Fig.7 g

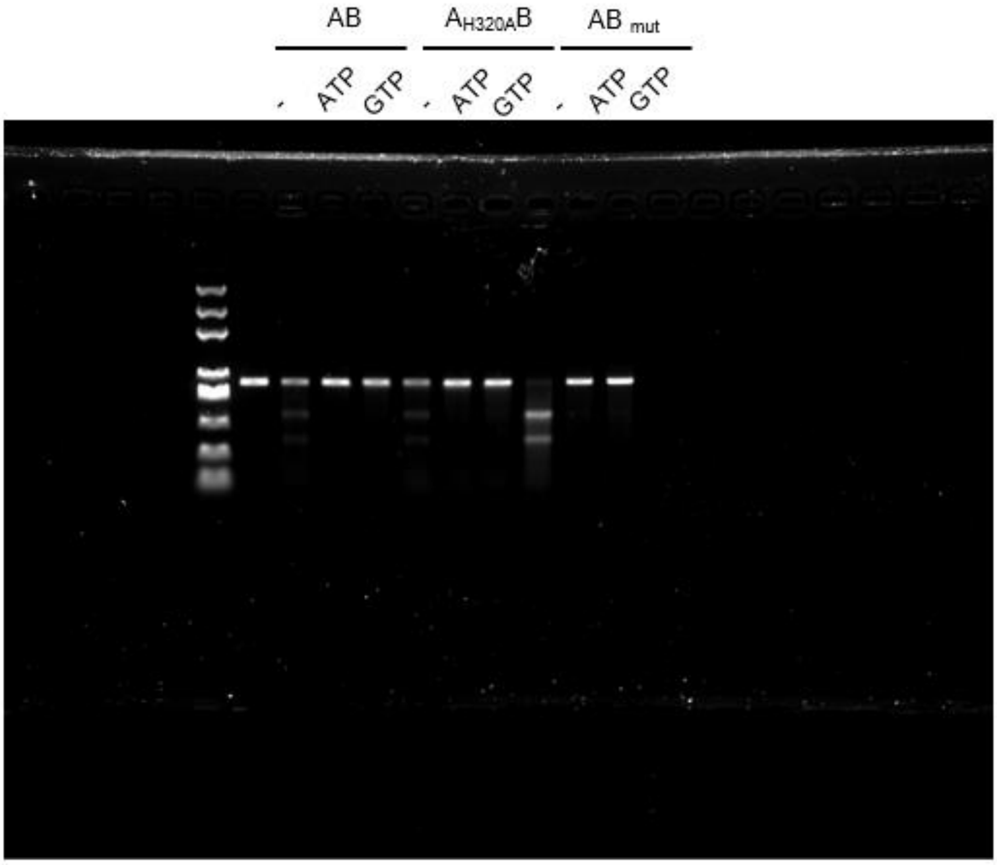

Supplementary Fig.7 h

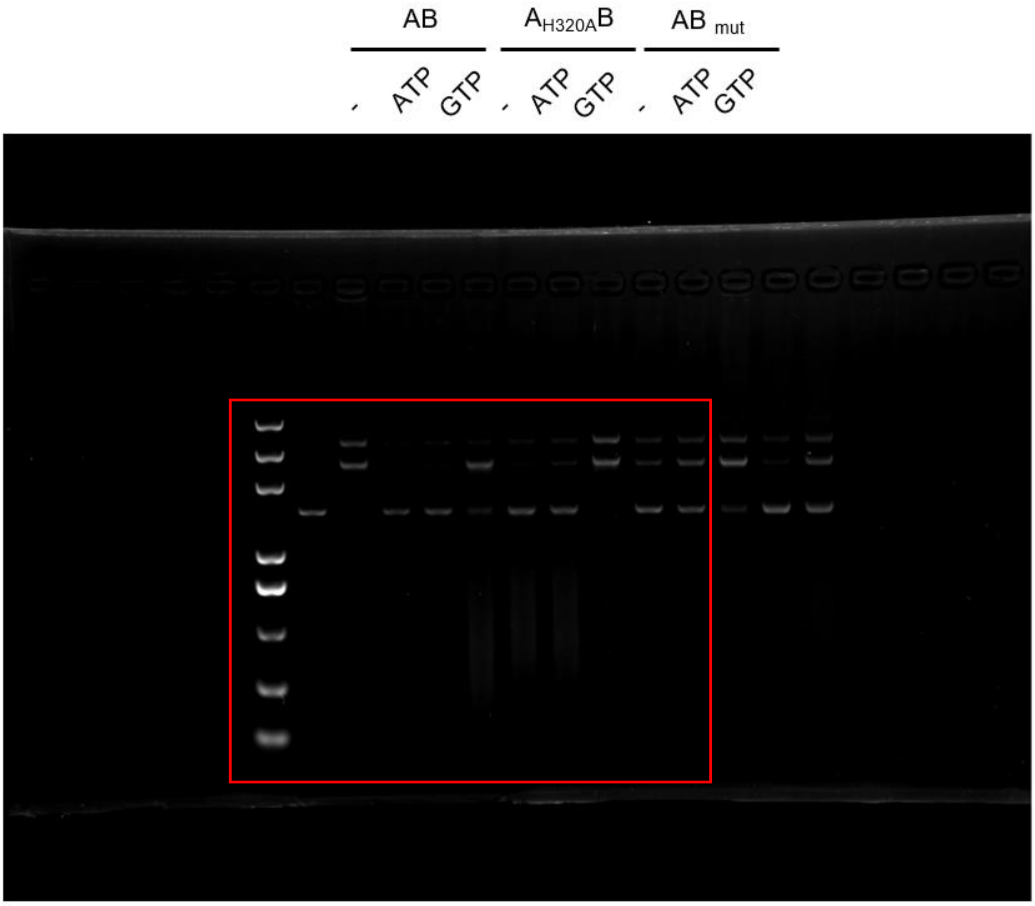

Supplementary Fig.9

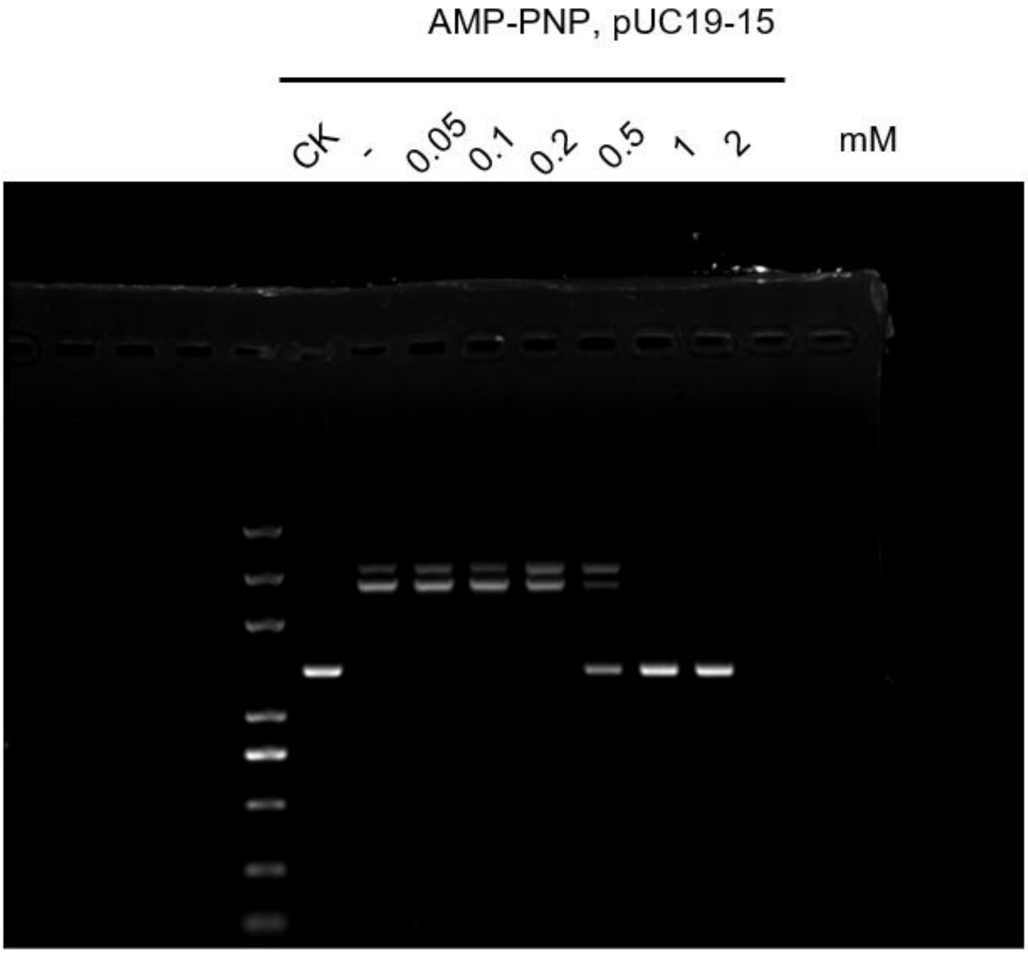

Supplementary Fig.11

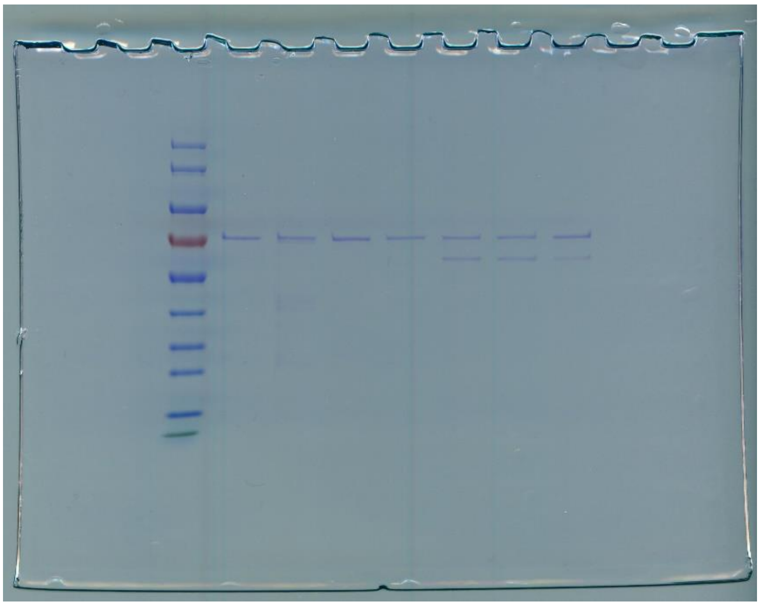

T2 phage plaque assay

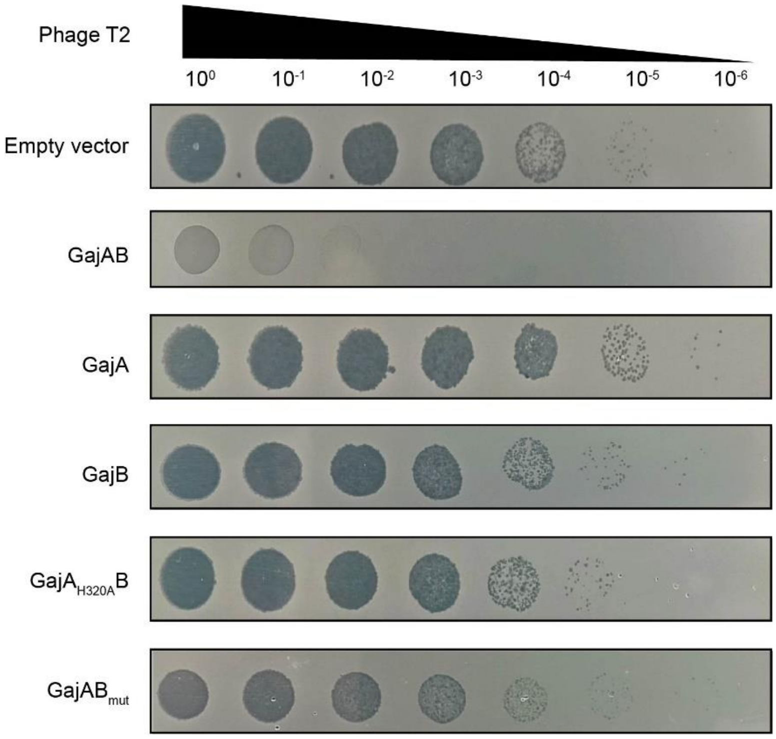

T2 phage resistance assay of E. coli expressing various Gabija genes and an empty control vector.10-fold dilutions of T2 phage solution infect E. coli harboring various Gabija genes and indicated mutant. Empty vector was used as a control. GajAB, wild type Gabija complex; GajAH320AB, Gabija complex with H320A mutation in GajA; GajABmut, Gabija complex with D162A and E163A mutations in GajB; GajA, the GajA gene alone; GajB, the GajB gene alone.

T2-Empty vector

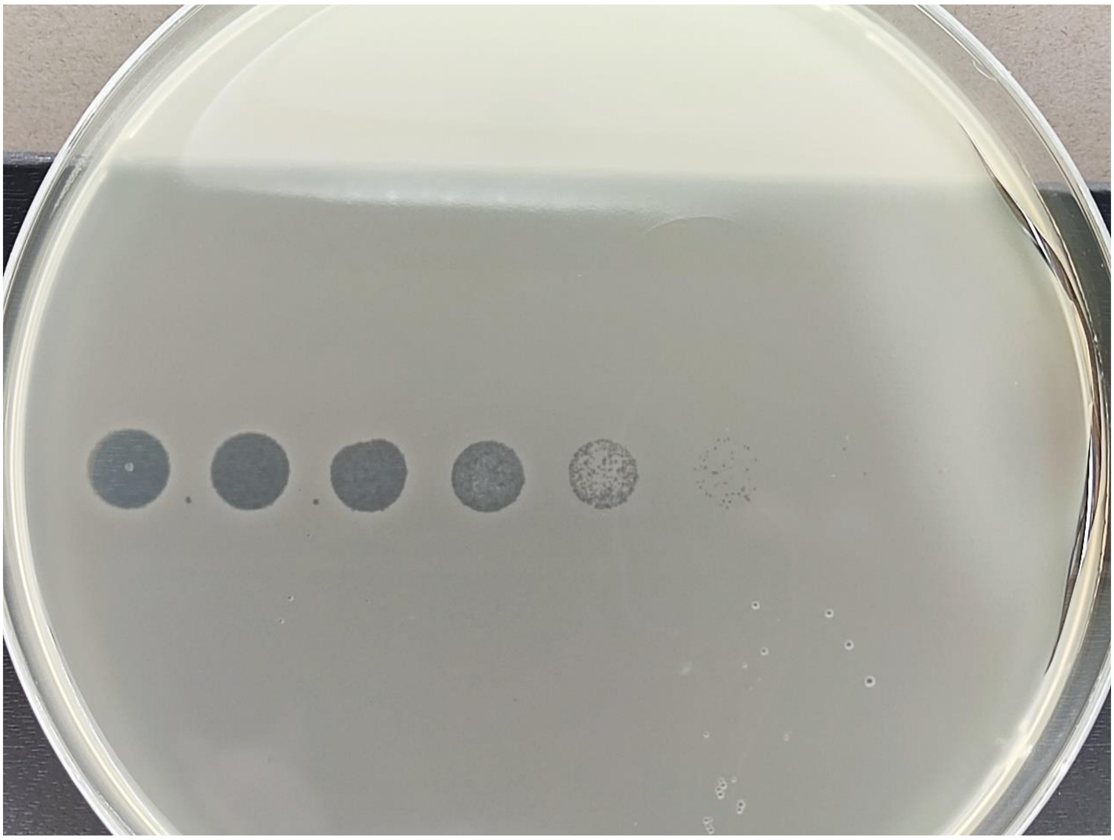

T2-GajAB

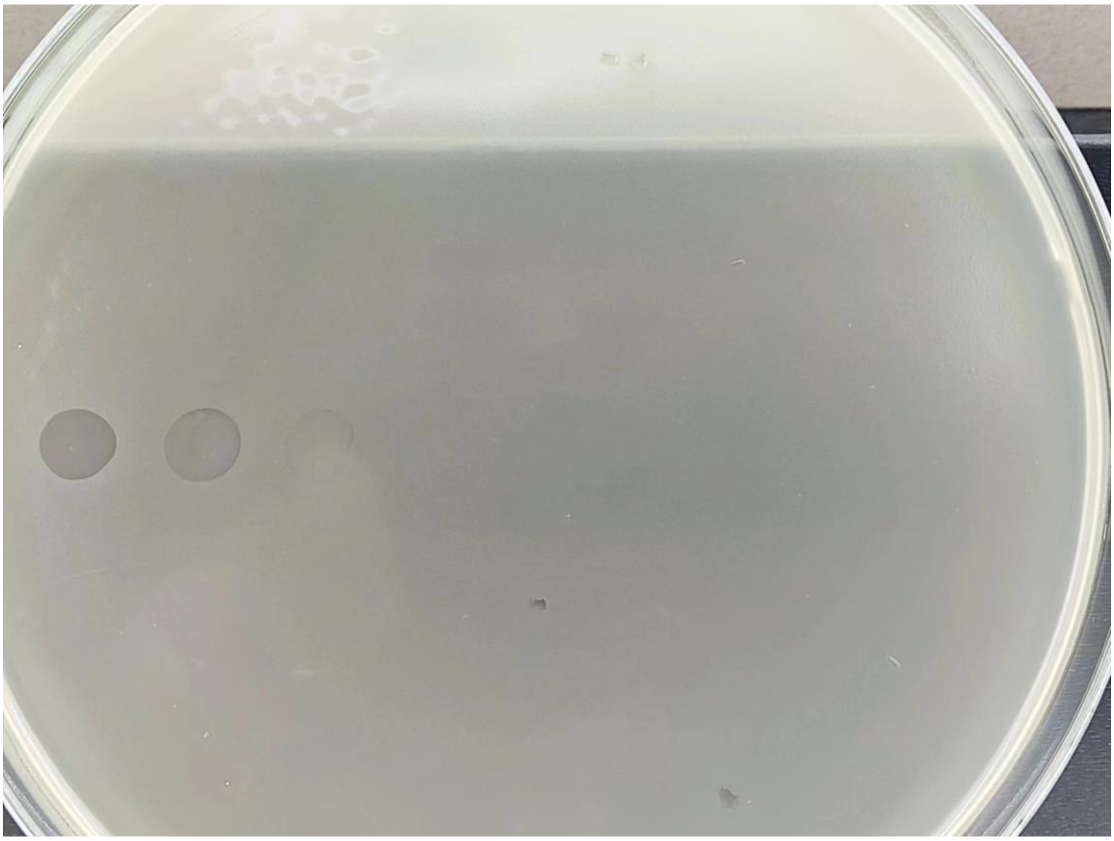

T2-GajA

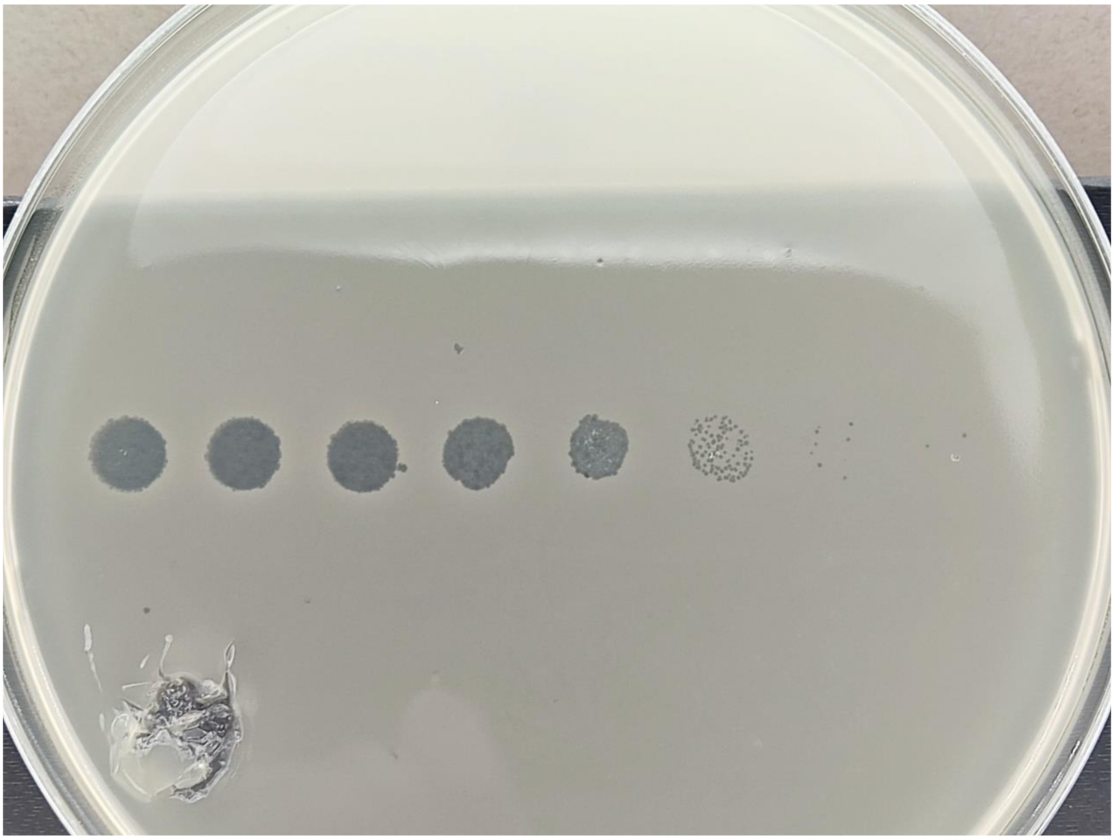

T2-GajB

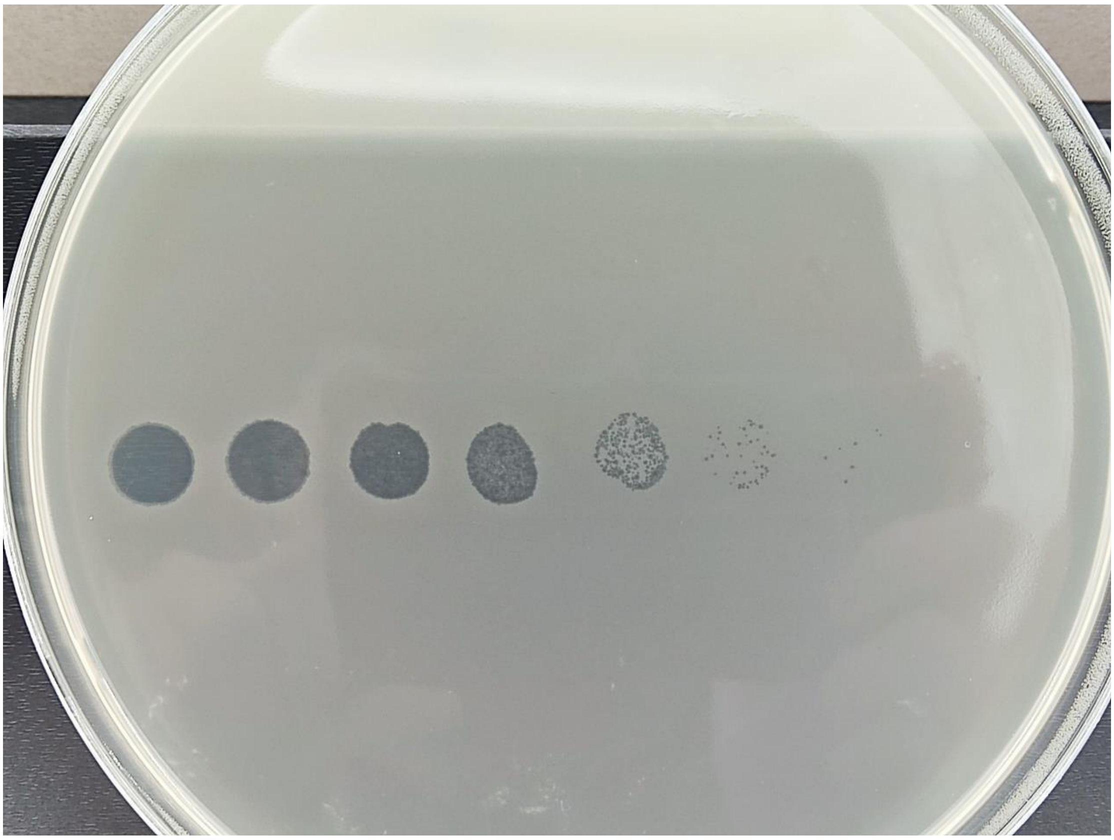

T2-GajA$_{H320A}$B

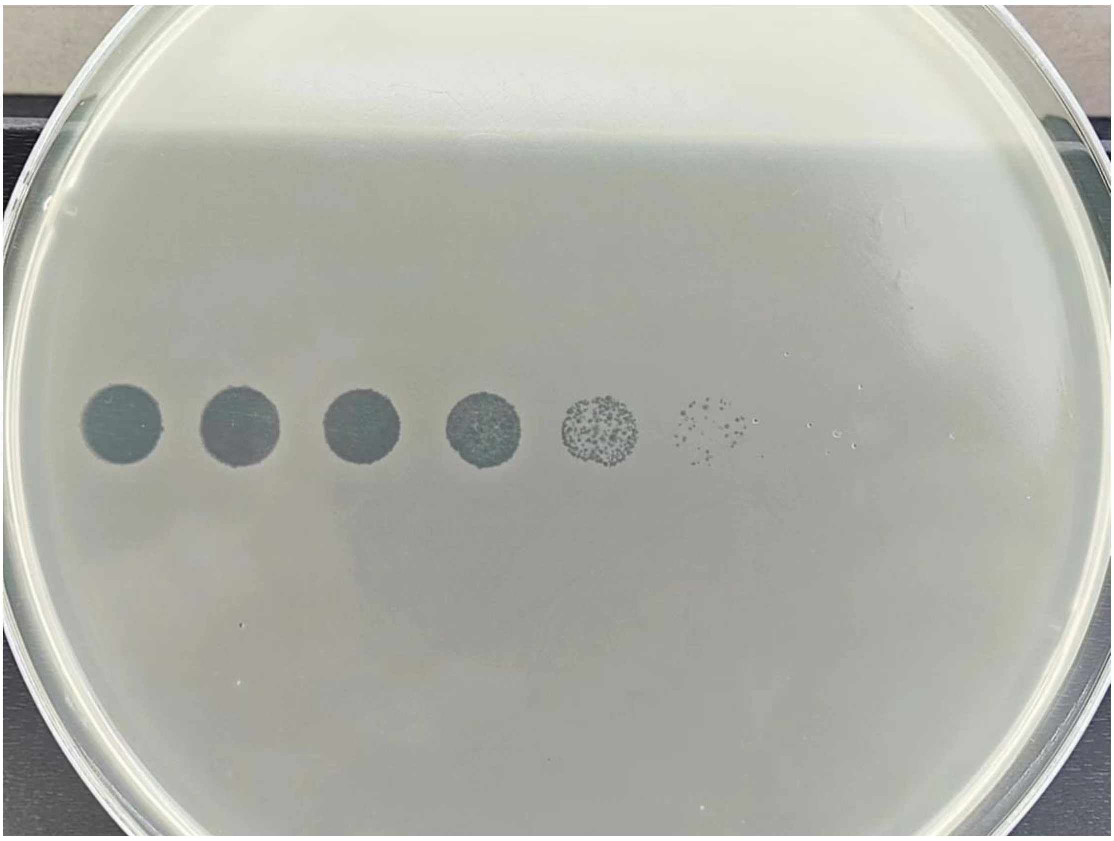

T2-GajAB$_{mut}$

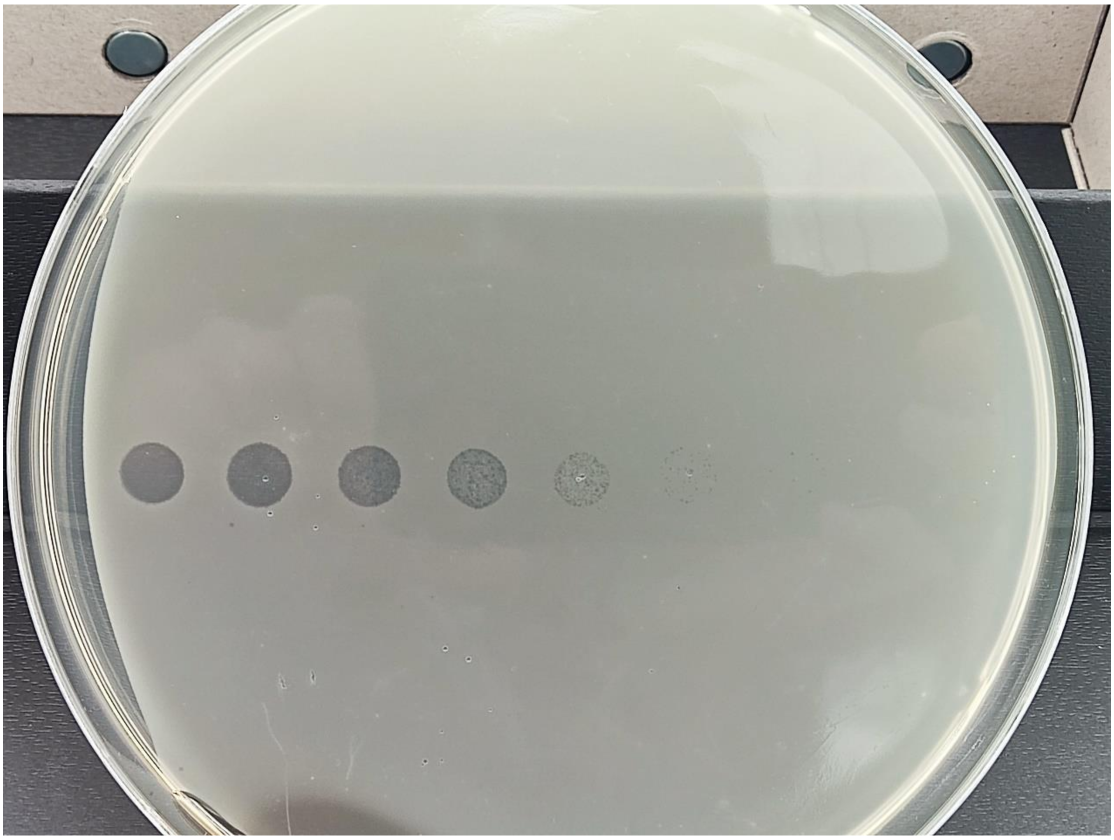

Supplement: Supplementary file 3 — Source Data [file 41467_2024_45173_MOESM3_ESM.zip › source data.pdf]
